# The salivary microbiome shows a high prevalence of core bacterial members yet variability across human populations

**DOI:** 10.1038/s41522-022-00343-7

**Published:** 2022-10-20

**Authors:** Xinwei Ruan, Jiaqiang Luo, Pangzhen Zhang, Kate Howell

**Affiliations:** School of Agriculture and Food, Faculty of Veterinary and Agricultural Sciences, University of Melbourne, Parkville, 3010 Australia

**Keywords:** Clinical microbiology, Next-generation sequencing, Dental conditions

## Abstract

Human saliva contains diverse bacterial communities, reflecting health status, dietary patterns and contributing to variability in the sensory perception of food. Many descriptions of the diversity of the salivary microbiome have focused on the changes induced by certain diseased states, but the commonalities and differences within healthy saliva have not been fully described. Here, we define and explore the core membership of the human salivary microbial community by collecting and re-analysing raw 16S rRNA amplicon sequencing data from 47 studies with 2206 saliva samples. We found 68 core bacterial taxa that were consistently detected. Differences induced by various host intrinsic and behaviour factors, including gender, age, geographic location, tobacco usage and alcohol consumption were evident. The core of the salivary microbiome was verified by collecting and analysing saliva in an independent study. These results suggest that the methods used can effectively define a core microbial community in human saliva. The core salivary microbiome demonstrated both stability and variability among populations. Geographic location was identified as the host factor that is most associated with the structure of salivary microbiota. The independent analysis confirmed the prevalence of the 68 core OTUs we defined from the global data and provides information about how bacterial taxa in saliva varies across human populations.

## Introduction

As a complex ecosystem, the human oral cavity hosts thousands of bacterial taxa, interacting with themselves, other microorganisms and the broader cavity^1^. It is an ecological system that contains many distinct sub-niches, including saliva, dental plaques, gingival sulcus and epithelial cells on the cheek, tongue and teeth^2^. High heterogeneity has been reported between the composition of microbial communities that colonise on different sites^3^. Saliva is recognised as a reservoir of microorganisms from all ecological niches in the human mouth with long-term stability^4^. The ensemble of microorganisms and the expressed genetic material in human saliva; the ‘salivary microbiome’ is crucial to understanding the healthy state of humans, and indeed, what this means for sensory appreciation of foods and beverages.

The structure of the salivary microbiome may be confounded by inter-individual variance. Characterising the microbial communities commonly found in most human saliva regardless of the study-specific variation could help establish the connection between salivary composition and food preference. Comparing results from different studies introduces significant technical and bioinformatic biases^5^, especially when studies have targeted different 16S rRNA hypervariable regions for amplification^6^. On this basis, the shifts from a common salivary microbiome by diseases or host lifestyle factors will also be more prominent. An integrated analysis of both sequencing data and associated metadata can summarise the existing knowledge and identify the commonalities and differences in salivary microbiota between people from various backgrounds.

There have been attempts to define the core oral microbiome of healthy humans^7^. The core microbiome is described as the common group of microbes that are important for host biological function^8^ and provides a foundation for prioritising members adapted to the host environment^9^. Although the shift in human salivary composition caused by diseases has been studied for decades^10–15^, our understanding of the impact of host intrinsic and behaviour factors is still limited.

Many host characteristics can impact upon the composition of the salivary microorganisms, including age^16^, diet^17,18^, ethnicity^19^, gender^20^, smoking^21^, alcohol use^22^, circadian rhythm^23^, body mass index^24^ and the type of stimulation^25^. Some studies have correlated the diverse microbiome with the distinct sensory responses between consumer groups^26,27^. It has also been reported that people from different countries are colonised with distinct salivary bacterial communities^28^. Li et al. analysed the human oral microbiome from Africa, Alaska and Germany and reported differences between the human groups living in various climate conditions^29^. However, a clear global pattern in salivary microbial composition is lacking, which considers both core microbial populations and variability amongst different groups.

In this paper, we collected raw 16S rRNA sequences of human salivary microbiota from 47 publicly available datasets spanning 15 different countries. These raw data were systematically re-analysed and pooled together to define a core salivary microbiome. We classified all sequences into operational taxonomic units (OTUs) at 97% identity against the expanded Human Oral Microbiome Database (eHOMD) to minimise the technical variation induced by comparing data from different hypervariable regions. It allowed us to make a comparison between studies and reduced the redundancy in the dataset for defining the ‘core’ microbial members. Using the metadata acquired with raw data, we also investigated the influences of several host factors and technical factors on human salivary microbiota. Factors that showed a potentially strong impact on shaping microbial communities in saliva were selected, and the taxa as potential biomarkers were identified, and linked with functional predictions. Finally, verification of the integrated study was sought through a targeted sampling effort. Here, saliva samples were collected from 26 healthy individuals and analysed for microbial composition to compare against the results found from the global dataset. These data confirmed the composition of the core microbiome members, but the verification of ethnic origin was not possible. Our study contributes to fundamental understanding of the stable and differential salivary microbiome across healthy adult populations.

## Results

### Inclusion of studies and sequences

**Fig. 1:**
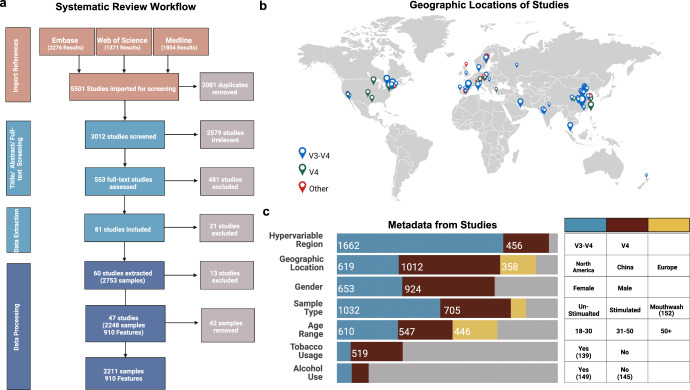
Literature search procedure and metadata of included studies. **a** Large-scale literature searching and data filtering process, followed by the number of samples submitted to the bioinformatic analyses; **b** The locations of studies, the scale of symbols that reflect the number of samples of each study; **c** Distribution of metadata categories. Created with BioRender.com.

In this study, we extracted the 16S rRNA gene amplicon sequencing data of healthy human saliva from 47 studies (Fig. 1a and Supplementary Table 2). We extracted data from subjects who had no diagnosed disease state, hereafter named ‘healthy’. Of course, subjects could have had subclinical diseases or may have altered health status for undisclosed reasons, but we considered that this would be true of the wider human population and, therefore, able to be included in our study. A total of 107,005,868 high-quality 16S rRNA sequences were obtained. After removing all samples below 2000 reads, 2206 samples with 909 OTUs were retained. The retained samples included studies from 15 countries (Supplementary Table 2). Most studies were conducted in three geographic regions: North America, Europe and China (Fig. 1b). Most sequences included in this study were generated from the hypervariable region ‘V3-V4’ and ‘V4’ (Fig. 1c).

### Intrinsic and lifestyle factors have a significant effect on the host salivary microbiome

**Fig. 2:**
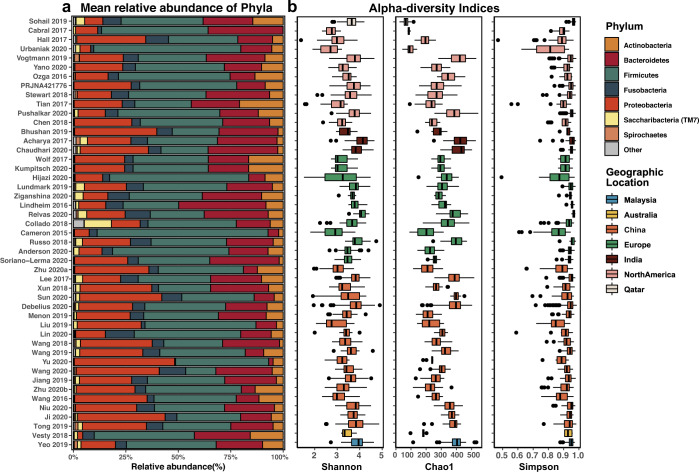
Taxonomic composition and alpha diversity of included studies. **a** The mean community composition of each study at the phylum level; **b** The alpha diversity measured by Shannon index; Chao1 index and Simpson’s index, the colour of boxes stand for the geographic location of the studies. The horizontal bars within boxes represent medians. The tops and bottoms of boxes represent the 75th and 25th percentiles, respectively. The whiskers represent ±1.5× the interquartile range. The other boxplots in this study follow the same definition.

Large variability between studies was observed in the number of reads, taxonomic profile and alpha diversity (Fig. 2 and Supplementary Fig. 1). Phylum *Bacteroidetes*, *Proteobacteria*, *Firmicutes*, *Fusobacteria* were dominated among all studies, while their proportion varied (Fig. 2a). When studies were grouped by the geographic locations they originated from (coloured in Fig. 2b), there is generally no difference between their intra-community diversity, represented by Shannon, Chao1 and Simpson indices. Only one study conducted in Qatar showed a relatively lower Chao1 index and higher Simpson’s diversity indices than studies from other locations. However, it is hard to determine whether such variation is caused by geographic location or other technical variations.

**Fig. 3:**
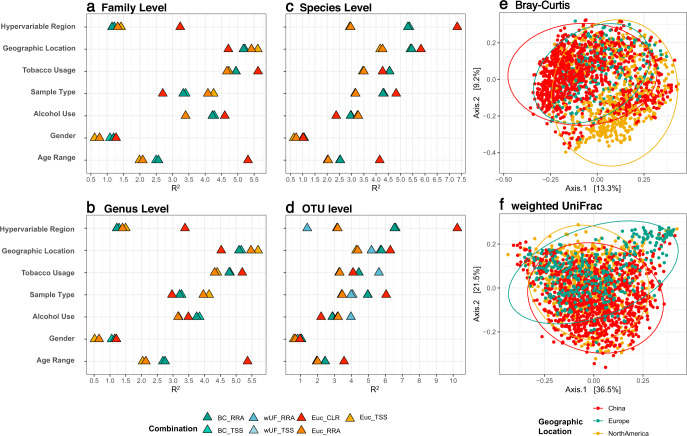
The variability in human salivary microbiota can be explained by different factors. Among them, hypervariable regions and geographic locations have the largest impact. The effect of the categories on the clustering of the sample was measured using PERMANOVA at four taxonomic levels: family (**a**), Genus (**b**), species (**c**) and OTU level (**d**). The colour indicates the different combinations of normalisation (TSS total-sum scaling, RRA rarefied relative abundance, CLR centred log-ratio) and indices (BC Bray–Curtis, EUC Euclidean, wUF weighted UniFrac). Because the results of rarefication (RAR) were very close to TSS and RRA, they were not displayed in the figures. Principal coordinate analysis (PCoA) with Bray–Curtis (**e**) and weighted UniFrac (**f**) showing the differences between samples from North America, Europe and China.

Because of the large disparity of methodologies amongst the studies used in our global analysis, we applied several different strategies for normalisation. When investigating the influences of different categories using permutational multivariate analysis of variance (PERMANOVA) tests, these normalising methods were combined as appropriate with different distance metrics, including Bray–Curtis, weighted UniFrac and Euclidean distance. Overall, the effect of rarefaction (RAR), total-sum scaling (TSS) and the rarefied relative abundance transformation (RRA) were very similar in the result of PERMANOVA (Fig. 3a–d and Supplementary Table 4).

Under all combinations, the beta-diversity analyses showed that all metadata categories measured have a significant (*p* < 0.001) effect on the bacterial profile of human saliva at all taxonomic levels, adjusted for the study effect (Supplementary Table 4). However, only limited variation among samples has been explained by these factors (*R*^2^ < 10%). Meanwhile, the ‘study’ also significantly explained the differences between samples, accounting for around 35% of the variability between samples. When using Bray–Curtis dissimilarity and Euclidean distance, the samples separated distinctly according to the hypervariable regions in PCoA plots, whereas the plot constructed using the weighted UniFrac revealed the clusters formed by the samples from different geographic locations (Supplementary Fig. 2). The result of PCoA analysis also showed there were significant differences between samples from three main geographic locations (Fig. 3f), with more than half (58.0%) of the variance explained by the first two dimensions, using weighted UniFrac distance. In contrast, the differences between locations were confounded by which hypervariable regions were sequenced in the PCoA plot for Bray–Curtis dissimilarity (Fig. 3e and Supplementary Fig. 2d–f). The results suggest that host intrinsic and lifestyle factors significantly influence the microbial profile in human saliva, regardless of the normalisation methods used and the variation induced by technical factors.

### A core microbiome is defined by saliva from healthy humans

**Fig. 4:**
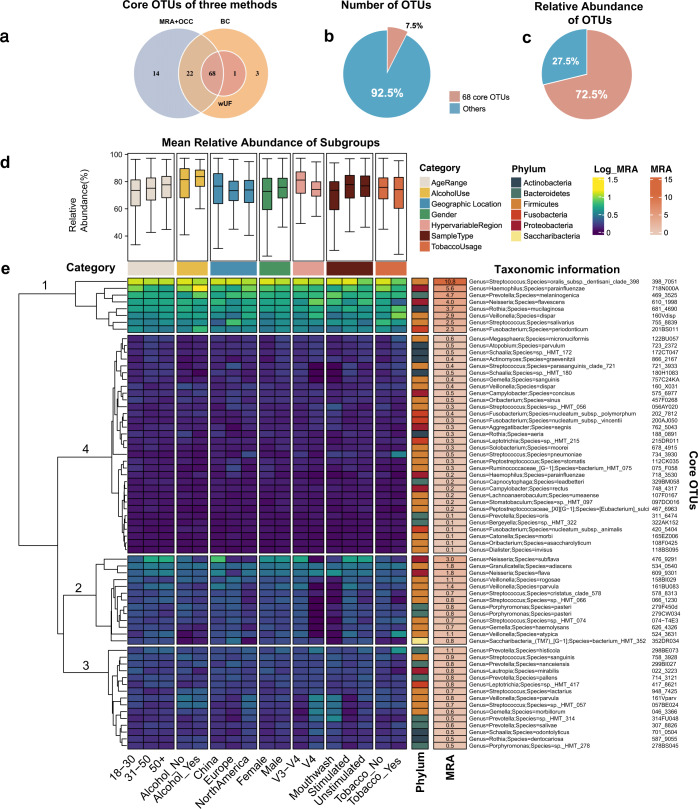
The core OTUs are defined by both the abundance and occurrence of the OTUs. **a** Venn diagram showing the interaction between three methods used to define the core. Sixty-eight OTUs were defined as the core for all methods. (MRA + OCC: The thresholds were set on mean relative abundance and occupancy to define the core; BC: The method adapted from Shade and Stopnisek using Bray–Curtis similarity; wUF: The method adapted from Shade and Stopnisek using weighted UniFrac distance). **b** Pie chart showing the number of the core (pink) versus other OTUs (blue) identified in percentage. **c** Pie chart showing the relative abundance of the core and other OTUs across all samples. **d** Relative abundance of 68 core OTUs across sub-groups classified by seven categories. **e** Heatmap showing the log-transformed mean relative abundance of each core OTU at each level of different categories.

Despite the large intra- and inter-study variability, many OTUs still showed a consistently high presence and relative abundance across studies (Supplementary Fig. 3). These persistent OTUs detected across studies with different protocols could be functionally important for the salivary microbiome of healthy adults. We wanted to identify the most widespread microbial taxa within a specific population that allows us to better understand the broad structure of microbiomes and their potential functional consequences^8^. The abundance and occurrence frequency of taxa are two important criteria used to define the ‘core salivary microbiome’. Conventionally, thresholds on these two parameters filter all taxa detected, and taxa that meet both criteria can be classified as the core salivary microbiome^9^. Recently, a more standardised procedure based on abundance-occupancy distribution was proposed^30^. We employed both strategies to identify the OTUs with high persistence in human saliva. To begin, the core OTUs were determined by filtering all OTUs based on mean relative abundance and occurrence frequency using the criteria described in the Methods^9^. In total, 11.6% of all OTUs (105 OTUs) were included as Core 1 (Fig. 4a: MRA + OCC; Supplementary Table 5). Meanwhile, according to the method proposed by Shade and Stopnisek^30^, OTUs were ranked depending on their occupancy across studies, and the contribution of top-ranked OTUs to beta diversity was expressed by Bray–Curtis similarity and weighted UniFrac distance. Two groups of the core microbiome were prioritised by these two indices, to give different inclusions in Core 2 (Supplementary Fig. 4; Supplementary Table 5), consisting of the top 69 OTUs (using weighted UniFrac; Fig. 4a: wUF) and 94 OTUs (using Bray–Curtis; Fig. 4a: BC).

Overall, 68 OTUs were shared across all three methods (Fig. 4a), accounting for 7.5% of all OTUs detected and 72.5% of all 16S rRNA gene sequences after clustering and filtering (Fig. 4b, c). *Firmicutes* account for nearly half (46.4%) of all core OTUs, while only one OTU belongs to *Saccharibacteria*. The mean relative abundance (MRA) of each OTU in sub-groups classified by different factors was also measured (Fig. 4d). On average, the core OTUs were highly prevalent (73.2 ± 3.4% of cumulative relative abundance) in saliva samples across different levels in sub-groups classified by age, gender, geographic locations, hypervariable regions, sample type, smoking and drinking habits. The core OTUs were clustered into four main groups based on their distribution pattern in sub-groups (Fig. 4e). The eight OTUs affiliated to Cluster 1 showed overall high abundance in all sub-groups. Cluster 2 consists of core OTUs with a slightly lower mean relative abundance than Cluster 1 and higher intra-group variability. Notably, although having a higher MRA than some members of Cluster 1, ‘476_9291’ was still classified as Cluster 2. The reason could be its biased presence in sub-groups. For example, the relative abundance of ‘476_9291’ is higher in samples from China than other two locations. The other two clusters contain OTUs with lower MRA than Cluster 1 and 2, while variations can still be observed within sub-groups.

We further applied a network analysis built by Spearman’s correlations to investigate whether the core OTUs defined were also important to the structure of a co-occurrence pattern. The resulting co-occurrence network contains 293 nodes and 1424 significant correlations (edges) (Supplementary Fig. 5). Although most of the core OTUs have relatively low connectivity, they associate with each other rather than with rare OTUs. Nine OTUs were identified as potential ‘hub’ OTUs based on their centrality and the number of links in the network (Supplementary Figs. 5, 6), and as such to community stability. Two core OTUs were identified as hub taxa; ‘122BU057’ (*Megasphaera micronuciformis*) and ‘524_3631’ (*Veillonella atypica*). Compared to other ‘hub’ taxa, they showed lower connectivity and relatively high betweenness centrality (Supplementary Fig. 6).

### Geographic location is the host factor that most associated with human salivary bacterial composition

**Fig. 5:**
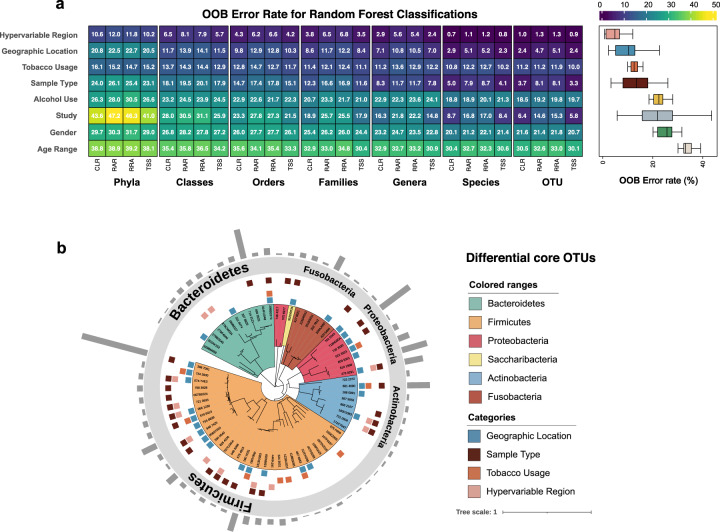
Salivary microbiome members significantly contribute to categorisation of metadata. Random Forest models showed the association of categories with the salivary microbiome and the core OTUs contributing to the accuracy of these models. **a** Error rate (%) for the random forest classifications conducted with samples grouped by eight different categories. **b** Phylogenetic tree indicates the taxonomic information of 68 core OTUs. The coloured squares between the tree and the annotation of phylum indicate the OTUs that were defined by the Random Forest model as ‘important’ for distinguishing between different levels in each category. The bars on the outmost ring show the mean relative abundance of each OTU.

To investigate which metadata category is most associated with the structure of the salivary microbiome, we established random forest models to link the seven categories described above and a new category, study, with the salivary microbiota data at seven taxonomic levels (OTU, phylum, class, order, family, genus and species). The effect of four normalisation methods was compared using the OOB error rate generated by random forest classification. In total, 224 random forest models were constructed (Fig. 5a). Among four normalisation methods, total-sum scaling produced the models that were, on average, the most accurate. Generally, the random forest models built with microbial communities at OTU levels have the lowest error rate (mean = 13.0%), while the models constructed at phylum levels have the highest (mean = 26.8%). The model built with the hypervariable region used for sequencing was also the category that showed the lowest error rate. Geographic locations demonstrated the second important association with bacterial communities, with the lowest error rate among biological factors. The random forest model constructed by the other two categories, sample type and tobacco usage, also showed a relatively lower error rate than other categories. The study constructed the models with high error rates at phylum (44.3 ± 2.5%) and class level (29.3 ± 2.1%). However, the error rate of models built with the study rapidly dropped with the increase of taxonomic levels, reaching 10.5 ± 5.1% at the OTU level. Gender and age range led to poorly performing models. To further validate the result, we built additional random forest models using the functions of another R package, *Caret*. The accuracy of using metadata categories to classify salivary microbiota data at seven taxonomic levels under only TSS normalisation was measured. In this method, five-fold cross-validation was performed; 75 and 25% of samples were used as training and test set. We found the resulting accuracy of the test sets was very similar to the results of random forest models constructed by *randomForest* () (Supplementary Fig. 7). Considering the ability of host factors to classify samples using the random forest model may be biased by hypervariable regions, we sub-grouped the samples by hypervariable regions, resulting in two large datasets (V3-V4 and V4). Within two datasets, geographic location consistently produces random forest models with high accuracy (tested by package *Caret*; Supplementary Fig. 8).

We wanted to determine whether the defined core microbiome could be used as biomarkers to differentiate people categorised by intrinsic and lifestyle factors. The random forest models showed low OOB error rates at OTU level were used (i.e. geographic location and smoking factors). The differential OTUs induced by hypervariable regions and sample types were also analysed to exclude the influence of technical factors. We further performed ten-fold cross-validation five times to measure the importance of OTUs used to train the model. All OTUs before the point that the cross-validation error curve starts to stabilise were defined as important OTUs. In total, we defined 59, 57, 34 and 70 important OTUs as biomarkers to differentiate samples according to geographic location, smoking habit, hypervariable region and sample type, respectively (Supplementary Fig. 9). Of these, 28, 10, 13 and 22 biomarkers were also classified as ‘core’ (Fig. 5b and Supplementary Fig. 10). Although 31 core OTUs showed the importance in discriminating samples according to geographic location and smoking, nearly half of them (15 OTUs) had the possibility of being confounded by technical factors (Fig. 5b). After excluding the OTUs that could be influenced by other factors, core OTU ‘322AK152’ (*Bergeyella sp.HMT_322*) was the OTU with the highest contribution to the classification of samples from three geographic locations. Meanwhile, ‘122BU057’ (*Megasphaera micronuciformis*) showed the highest importance among the core differential OTUs specific to smoking, followed by ‘524_3631’ (*Veillonella atypica*). We were surprised to find that these two OTUs were the only two OTUs that were defined as hub taxa in the co-occurrence network analysis (Supplementary Fig. 6).

### The salivary microbiota as biomarkers to differentiate Chinese and Western participants

**Fig. 6:**
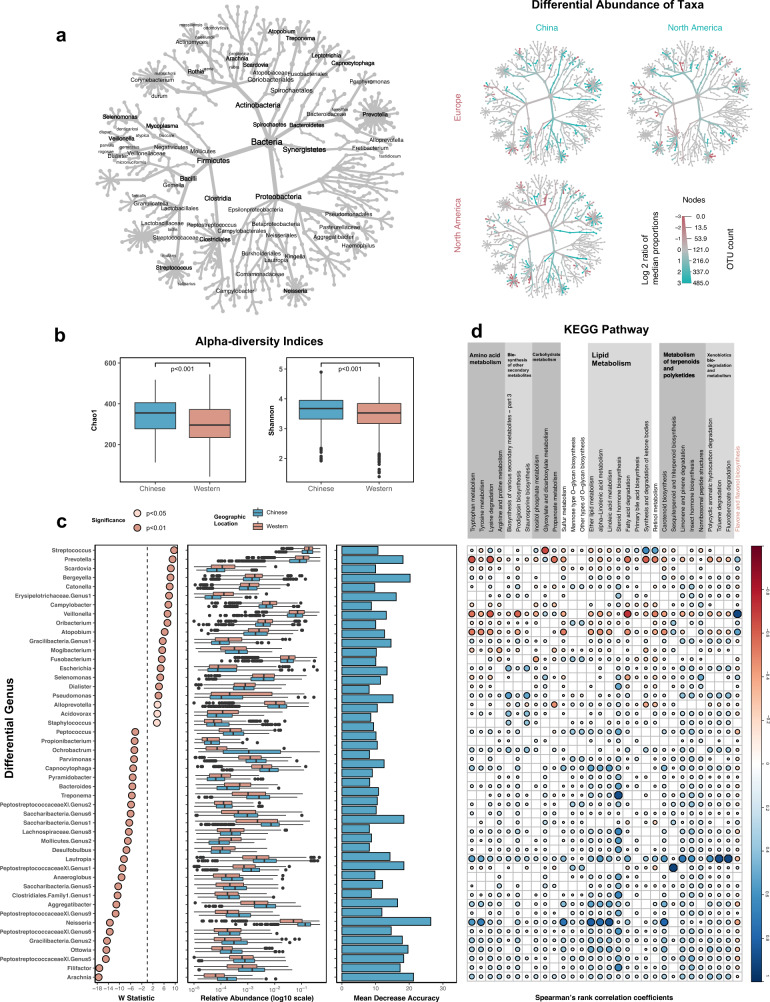
Distinct microbial profiles are evident in the saliva samples from Chinese and Western adults. **a** Taxonomic hierarchies show the relative enrichment of taxa in three geographic locations at phylum through species level. Coloured nodes indicate a log2-fold increase in the median abundance of the group in the x-axis (pink) or y-axis (blue). Only taxa showed significant changes (false discovery rate-adjusted Wilcoxon rank-sum *q* < 0.05) are displayed. **b** Comparison of salivary microbial alpha diversity between the Chinese and Western samples, calculated by Chao1 (*p* < 0.001, Wilcoxon rank-sum test) and Shannon index (*p* < 0.001, Wilcoxon rank-sum test). **c** Differential abundant genera identified between saliva from Chinese and Western samples. The panel on the left indicates the standardised effect sizes (W statistic) estimated via the difference in relative abundance using ANCOM-BC (taxa enriched in Western samples have a value shifted to the right, whereas taxa enriched in Chinese samples have a value shifted to the left); The panel in the middle shows the relative abundance of selected genera; the panel on the right indicates the Mean Decrease Accuracy of the random forest model established. **d** Spearman’s correlation coefficients were calculated between each pairwise comparison of differential genus and KEGG pathway. Only significantly correlated comparisons (*p* < 0.01, FDR-adjusted Spearman’s rank correlation) are displayed. The only Western-enriched pathway is marked in pink.

We further analysed the potential effect of geographic locations in higher taxonomic hierarchies, where many differences have been revealed. Of particular interest were taxa under phylum *Synergistetes* and *Spirochaetes*, Class *Mollicutes* and *Betaproteobacteria*, Family *Clostridiales* and genus *Prevotella*. Interestingly, we found many taxa not only differentiate Chinese samples from North American samples, but also differentiate Chinese samples from European samples (Fig. 6a). It suggested that the variance induced by geographic locations may be dominated by the differences between samples from Chinese and Western people. Therefore, we combined samples from North America and Europe into a single group, ‘Western.’ Compared to the Chinese grouping, the Western group has shown the significantly lower Chao1 and Shannon indices (Wilcoxon rank-sum test, *p* < 0.001; Fig. 6b), which may suggest a lower within-sample diversity (alpha diversity) of Chinese population than Western population. Next, we examined the differences between Chinese and Western in the salivary microbiota at the genus and species level (Supplementary Tables 6, 7). Besides establishing a random forest model, we also identified differential taxa using ANCOM-BC, adjusting for the hypervariable region. We found 48 genera identified as significantly different by both methods (Fig. 6c and Supplementary Table 6).

Finally, we performed the functional prediction-based 16S rRNA gene profiles to investigate the potential relationship between the differences in the salivary microbiota of the two groups and its function. Two methods, ANCOM-BC adjusted for the hypervariable region, and random forest model were used to identify which pathways were differential between Chinese and Western. The result of ANCOM-BC indicated that 69 pathways related to metabolism were differentially abundant between the two groups. The random forest classification model established using KEGG pathways demonstrated an unbiased error rate of 10.01% and revealed 46 differential pathways. Among them, thirty pathways belonging to nine upper pathways (level 2) were simultaneously defined by two methods as differing in abundance between Chinese and Western (Fig. 6d and Supplementary Table 8). A variety of pathways was in higher abundance in Chinese samples. The enrichment of these pathways in Chinese samples was mainly associated with the increased abundance of *Neisseria* and *Lautropia* and the depleted abundance of *Prevotella*, *Veillonella* and *Atopobium*. Notably, three lipid metabolism pathways enriched in Chinese samples, including ‘Ether lipid metabolism’ (ko00565), ‘alpha-Linolenic acid metabolism’ (ko00592) and ‘Linoleic acid metabolism’ (ko00591), have the highest standardised effect size (W statistics, Supplementary Table 8). The enrichment of these pathways related to lipid metabolism has been positively associated with the higher abundance of *Neisseri*a in Chinese (Fig. 6d). *Neisseria* may have also contributed to the pathway ‘Carotenoid biosynthesis’ (ko00906). Another metabolic pathway related to the metabolism of terpenoids and polyketides, ‘Sesquiterpenoid and triterpenoid biosynthesis’ (ko00909), showed a positive correlation with a genus belonging to *Peptostreptococcaceae*. In contrast, only one pathway named ‘Flavone and flavonol biosynthesis’ (ko00944) was enriched in the saliva samples from Western participants. A strong positive correlation has been demonstrated between this pathway and the increased abundance of *Veillonella* in the samples from the Western grouping.

### Validation of the core in an independent Australian cohort

**Fig. 7:**
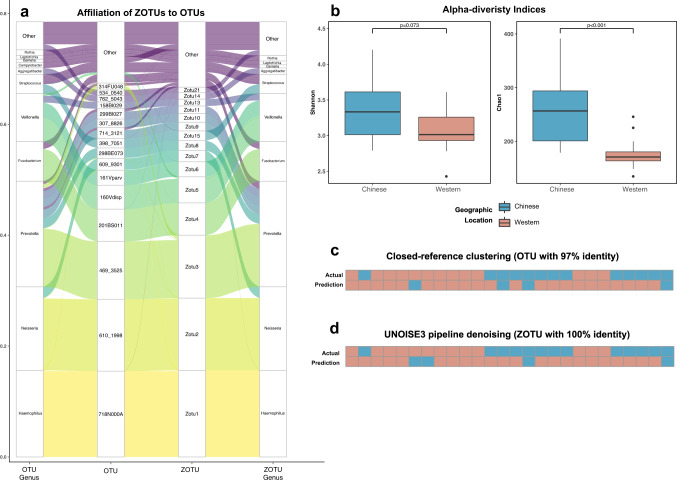
An independent cohort verifies the definition of core microbiome membership but cannot classify based on cultural background. **a** Alluvial plot showing the affiliation of ZOTUs to their originating core OTUs defined in the integrated analysis. **b** Comparison of salivary microbial alpha diversity between the Chinese and Western samples, calculated by Shannon (*p* = 0.073, Wilcoxon rank-sum test) and Chao1 index (*p* < 0.001, Wilcoxon rank-sum test), using the ZOTUs generated from denoising. **c**, **d** The prediction of the cultural backgrounds of the samples according to the random forest classification model constructed using the genus profiles of samples in the integrated analysis. The genus-level profiles of samples processed by **d** closed-reference clustering with 97% sequence identity and **e** UNOISE3 denoising with 100% sequence identity were used as the test set.

To validate the prevalence of the core OTUs in human saliva, we collected saliva samples from 13 Chinese and 13 Western participants and sequenced the extracted DNA with 515F-806R primers. In total, 841,188 high-quality 16S rRNA sequences were obtained, which clustered into 397 OTUs with 97% identity to the eHOMD database. Among them, the core OTUs we defined in the integrated analysis showed high relative abundance (78.3 ± 6.9%) in all collected samples. To increase the accuracy of the OTU assignment, we denoised the sequencing reads using the UNOISE3 pipeline and generated ZOTUs with 100% sequence identity. After re-clustering the core OTUs defined in the integrated analysis to ZOTUs, we observed that 59 of the identified OTUs in this independent dataset consisted of 87 ZOTUs, and thus made up close to 80% of the relative abundance (Fig. 7a). Although some sequences belonging to the same ZOTU are clustered to different OTUs, all the core OTUs contain at least one highly abundant ZOTU. The taxonomic profiles of the global core annotated by the eHOMD database and the ZOTUs annotated by the SILVA database were very similar at the genus level (Fig. 7a).

Using this independent dataset, we also wanted to verify whether differences we observed in the integrated analysis between the OTUs in saliva samples from Chinese and Western residences can also be found between those who were born in China and Western countries. Although the two groups did not differ significantly when considering the Shannon diversity index (Wilcoxon rank-sum test, *p* = 0.073; Fig. 7b), the Chinese group showed a higher Chao1 Index than the Western group (Wilcoxon rank-sum test, *p* < 0.001; Fig. 7c), which is in agreeance with differences in alpha diversity between Chinese and Western population of the integrated analysis. This result has been observed in analyses using OTUs (Supplementary Fig. 11) and ZOTUs (Fig. 7b). Meanwhile, no significant differences were observed between the two groups by beta-diversity analyses (Bray–Curtis and weighted UniFrac distance, Supplementary Table 9), which is compatible with the homogeneity of microbial compositions within the sample geographic location. We used the random forest classification model constructed using the genus-level profile of the large-scale dataset to predict the Chinese and Western samples in this independent study. This dataset’s genus-level relative abundance table was prepared from both the OTU table with 97% identity to the eHOMD v15.1 database (Fig. 7c) and the ZOTU table annotated by the eHOMD v15.22 database (Fig. 7d). The accuracy of both predictions was relatively low, with 57.7% (AUC = 79.88%) for the OTU table and 50% (AUC = 77.51%) for the ZOTU table. The relatively high AUC values are likely a product of the very high true negative rate, which means that the models are only very good at predicting Western samples. Interestingly, most Western samples were correctly classified, while most samples from Chinese participants were classified as being ‘Western’ in this analysis (Fig. 7c, d).

## Discussion

There is ample knowledge on the differences between salivary microbiota from the oral cavities of healthy and disease-affected individuals^11–13^, yet our perspectives on the commonalities and variabilities in healthy salivary bacteria remain limited. Our systematic selection of studies, together with the re-analysis of the 16S rRNA amplicon sequencing data from 47 studies, offers a description of the salivary microbiome presented in adults without diagnosed disease. Our study has defined the core members of salivary bacterial communities across 2206 samples from 47 studies and used metadata captured in these studies to investigate the role of different intrinsic and extrinsic factors on the occurrence of these core. It is clear that core members of the microbiome differ between geographic locations of collected saliva, and our analysis shows that Chinese participants are different from Western participants (encompassing European and North American studies). A prediction of the pathways enriched in each collective indicates that bacterial metabolic pathways are likely to influence the aroma and flavour perception of foods. These results show that despite the core microbial members of saliva being common across humans, there are differences, likely due to host factors.

Based on the abundance and occurrence frequency of the OTUs, the definition of core microbiome highlighted the persistent and conserved microbial communities in human saliva across the globe. Here, we compared approaches adapted from two studies^9,30^ to define the core. The method adapted from the study of Wu et al.^9^ is a relatively conventional strategy that has been chosen by many other studies with customised thresholds^31–33^. For another approach we applied in this study^30^, we determined the occupancy of OTUs according to their detection over study. When evaluating the contribution of top-ranked OTUs in occupancy to the beta diversity of the community, we further used weighted UniFrac due to its effectiveness in minimising the biases induced by the selection of hypervariable regions.

The resulting core members identified by these methods have a lot in common. A majority of the core OTUs defined by Shade’s method is also included in the cores defined by Wu’s method, suggesting the recently developed multi-step approach is effective in determining taxa with high prevalence. The general high relative abundance of the core across different sub-groups emphasised the utility of this pipeline in identifying the persistent members across diverse datasets. Most of the core salivary microbiota we defined had been proposed in previous studies as prevalent bacteria in the human oral cavity that persistently span across different individuals^7,34–36^. The dominant genus of the core we defined, *Streptococcus*, *Neisseria*, and *Prevotella*, were identified as the core human salivary microbiome in a recent study based on MG-RAST data^37^. Ten OTUs belonging to genus *Streptococcus* were included in the ‘core’, two of which were classified to the cluster with overall the highest relative abundance across all dimensions. The prevalence of *Streptococcus* we observed is consistent with a previous study defining the healthy core from the 454 pyrosequencing results of three individuals^7^. The most abundant core OTU we found, *Streptococcus oralis* subspecies *dentisani*, has been documented in previous studies as potential oral health-promoting organism and is highly abundant in various oral niches of healthy humans^38,39^.

We further conducted a co-occurrence network analysis to investigate the role of these core microbiota in shaping the microbial community and found the co-existence between many members of the core (Supplementary Fig. 5). The presence of rare OTU that became the hub suggests that although some taxa are not persistently detected across the community, they may still be important for the overall structure of the salivary microbiome. However, many studies have shown that using Spearman’s correlation to analyse compositional data, such as sequencing data, is likely to generate spurious correlations^40–42^. Therefore, the results of co-occurrence network analyses should be interpreted with caution. It has been proposed that the oral microbiota of healthy individuals is both homoeostatic and dynamic^34^. The core microbial members consistently present in human saliva identified here may explain the stability of oral microbiota to some extent. We conducted an independent study to verify the prevalence of the core defined in the published studies. The high prevalence of the original core OTUs in this independent cohort suggests that the core human salivary microbiome we defined can be applied to different datasets. After re-clustering the core OTUs to ZOTUs with 100% sequence identity, we may conclude that the same members constitute the core, even if a different taxonomic resolution is applied.

Besides the core microbiome, there are ‘variable’ microbiota in human microbial communities, which vary among individuals because of unique lifestyle and genetic factors^43^. We performed analyses for beta diversity of samples (Fig. 3) and random forest classifications (Fig. 5a), demonstrating several factors-both technical and physiological-significantly discriminated between sub-populations. Because of the high heterogeneity between studies in their methodology, large inter-study variability was the main factor that affected the observed salivary microbiota^44,45^. For example, the impact of chosen hypervariable regions for sequencing on driving microbial community structures has been confirmed by our study. Inter-study variation may also be attributed to the criteria for recruiting participants. Although all samples included in this integrated analysis were collected from the control groups and populations without specific diagnosed diseases, the definition of ‘healthy’ varies. For example, the use of antibiotics was not always considered as an exclusion criterion, and when included, different time intervals were adopted. However, we aimed to construct a microbial community that reflects the salivary microbiota of real-life consumers.

The importance of various host intrinsic and lifestyle factors, especially smoking, changed the salivary microbial composition^46^. We identified a core OTU belonging to *Megasphaera micronuciformis* as a biomarker for reported smoking^47,48^. A *Veillonella atypica* OTU vary between smokers and non-smokers, agreeing with a previous study^49^. Another strong determinant of the salivary microbiome we defined was ‘sample type’. As we found in this study, the difference between the mouthwash sample and the other two collection methods is greater than the difference between the stimulated and unstimulated saliva. Contrary to the result of Jo et al.^25^, the OTU belonging to *Neisseria flava* has not been identified as differential taxa for the type of saliva. The microbial composition of alcohol drinkers was different to non-drinkers, but small sample size hinders other conclusions.

Geographic location has been identified as the host physiological factor with the largest association on salivary microbiota (Fig. 5a). Although it only explained limited variability between samples’ microbial profiles, the observed variations were robust to the heterogeneity induced by different hypervariable regions used (Fig. 3e, f). To date, little is known regarding the influence of geographic locations on the human salivary microbiome. A comparative study reported the differences in saliva microbial composition in Alaskans, Africans and Germans^29^. Our result extends this study to demonstrate that core OTUs may differentiate saliva samples from North America, Europe and China. Given the high abundance and occupancy frequency of the core microbiome, we would expect these taxa to be effective indicators to predict the geographic background of saliva donors. We suggest that informative and complete metadata is needed to make claims across studies; our observations of differing alpha diversity amongst Chinses and Western populations would need to be verified in the absence of metadata from the studies investigated here.

The classification of Chinese and Western people in this study is based on cultural and geographic differences. China, a country located in East Asia, is usually classified as a non-Western country^50,51^. Meanwhile, countries from North America and Europe are usually defined as Western countries shaped by Western European culture^52^. Our study found a difference between saliva from Western and Chinese people in the abundance of *Veillonella* spp. Here, we showed that *Veillonella* was generally higher in Western samples. Such differences may further influence the flavone and flavonol biosynthesis pathways in the oral cavity. Our previous study revealed that Western-born and Chinese-born wine experts who live in Australia have different responses to the flavour of wine^53^. Since flavonol is a well-known constituent of wine related to the bitterness and astringency perception^54^, we would hypothesise that the enrichment of *Veillonella* in Western may affect their sensitivity to the phenolic compounds in wine. Regular consumption of flavonoid-rich foods, such as oolong tea, may increase the abundance of *Veillonella* spp. in human saliva^55^. These could link the differences between Chinese and Western groups in sensory evaluation and their salivary microbiota together. However, as a bioinformatics tool for amplicon-based prediction, PICRUSt2 is limited by the substantial intra-variation in genomic content^56^. Therefore, the inferred metabolic pathways reported here should be interpreted conservatively. Further investigations with higher accuracy, such as shotgun metagenomic sequencing, are required to verify the predicted functional differences. The contribution of the salivary microbiome to sensory perception of foods and beverages is a topic that received increasing interest yet is largely unexplored^57–59^. Our findings here give the new potential for further studies to understand the food preferences of different consumers, thereby facilitating food and beverage design.

When verifying whether the differences we observed in the integrated analysis can also be found in Chinese-born and Western-born people living in the same geographic location, the independent dataset we collected did not show differences between samples taken from Chinese and Western participants. It could be attributed to the technical variance induced by the different methodologies. Although the DNA extraction method, variable region amplified and processing protocol we used for validation were commonly chosen by studies included in the integrated analysis, we cannot completely exclude the possibility that the 16S rRNA gene processing and sequencing process may have generated variability that affected the results. It highlights the importance of promoting globally standardised saliva extraction and sequencing protocols such as the Earth Microbiome Project protocols. Notably, the two data analysis pipelines we conducted on the raw sequences led to similar results of diversity tests, which may suggest the data analysis methodology is not the main driver. The other reason for the difference between the results of the integrated analysis and validation could be the population-based variability. The results of random forest classification may lead to some interesting hypotheses. The prediction of models revealed that most of the Chinese samples in this cohort were classified as Western. The donors of these samples were wine experts, of Chinese ethnicity, born in China and living in Australia for no more than 18 months. It has been previously demonstrated that people in China is currently under the westernisation of lifestyle, with the associated shifts in microbial community composition^60,61^, especially in gut microbiota^62^. Meanwhile, recent studies reported that immigrants from Asia to western countries experience a ‘Westernisation’ of gut microbiota induced by dietary acculturation^63,64^ We hypothesise that such a phenomenon may also happen in salivary microbiota. In this study, we found the immigrants from China to Australia have not been predicted as ‘Chinese’ using the random forest model we trained using Chinese residents. It may suggest that lifestyle factors, such as dietary patterns, are more important determinants than ethnicity in shaping the salivary microbiota of the participants, leading to variation among different geographic locations that we observed in the integrated analysis. Furthermore, although the westernisation process in the lifestyle is ongoing on Chinese population around the world, the degree of westernisation could be different between local Chinese and Chinese immigrants who live in western countries. However, as no diet-related information has been included in this study, further analyses are required to investigate this hypothesis.

In summary, we have defined a core bacterial community in saliva from healthy humans, and this core demonstrated both stability and variability among populations. The prevalence of the core members of the saliva microbiome has been confirmed in an independent cohort. We have revealed the influence of various host factors, such as geographic locations and incidence of smoking and drinking, on the salivary microbiome. We also identified microbial and functional biomarkers to differentiate the Chinese and Western people, underlying the potential relationship between salivary microbiota and sensory perception. Results in this work will provide foundational information to inform future studies to understand the similarities and differences in saliva microbial composition, potentially associating oral to aroma and flavour perception of foods.

## Methods

### Literature search and data collection

To acquire sufficient data from healthy human saliva, available public studies related to human salivary microbiota were systematically reviewed. A literature search was performed using the combination of relative terms in EMBASE, MEDLINE and Web of Science for the studies published before November 2020 using the terms described in supplementary data (Supplementary Table 1). A supplementary dataset search in NCBI’s Sequence Read Archive (SRA) was also performed using the search term ‘salivary microbiome’. The resulting studies were screened according to the criteria. The included studies met the following criteria: (1) Having samples from participants without any diagnosed disease state. For studies investigating the influence of certain kinds of disease on salivary microbiota, only samples collected from healthy controls were included in the further analysis; (2) Using whole human saliva collected by spitting, swab, mouth washing or oral rinsing, samples exclusively extracted from any specific oral spot, like tongue surface, parotid gland, supragingival plaque, were excluded; (3) Using 16S rRNA gene high-throughput sequencing and sequenced with the Illumina MiSeq platform; (4) Having sequence files with quality score and associated metadata, information about geographic locations are required; (5) Having freely available sequencing data; (6) Sequencing data correctly separated according to the metadata.

Except for studies conducted in three main geographic regions, including North America, Europe and China, other studies were marked as from region named as ‘others’. Samples from studies targeted at ‘V1-V2’ or the ‘V4-V5’ region were classified as ‘others’ (Fig. 1c and Supplementary Table 3). Similarly, saliva samples collected by unconventional methods like ‘swab’ were also classified as ‘others’. People who recorded a smoking habit, whether they smoke e-cigarettes or tobacco, were categorised as smokers. Similarly, people who drink alcohol, regardless of the frequency or the type of alcohol consumed, were classified as drinkers. The given age was treated as a categorical factor, classifying into ‘18–30’, ‘31–55’ and ‘56+’. The samples without associated information to a particular category or classified as ‘others’ were excluded from downstream analyses related to the impact of this category. For example, the following analyses measuring the effect of hypervariable region on microbial profiles included only comparisons between the V3-V4 and V4 regions (Fig. 1c).

Raw sequence data acquired from the healthy individuals of selected studies were downloaded from SRA and European Nucleotide Archive (ENA), using SRA Toolkit. The files were converted to the FASTQ formats if necessary. Sequence data from each selected study were processed separately using QIIME2 (version.2020.2)^65^. The resulting OTU table was exported into BIOM format. Further analyses were carried out in R (version 4.1.0) with custom scripts.

### 16S rRNA gene sequence processing in QIIME2

Sequences with primers were trimmed with ‘q2-cutadapt’ (https://github.com/qiime2/q2-cutadapt) to retain the targeted hypervariable regions. The demultiplexed paired-end sequences were firstly joined by ‘q2-vsearch’ (https://github.com/qiime2/q2-vsearch), then subjected to a quality filter with a minimum quality of Q30. The remaining reads were then clustered into operational taxonomic units (OTUs) at 97% similarity against the expanded Human Oral Microbiome Database (eHOMD) version 15.1^66^ by the closed-reference OTU picking command. Reads that failed to match a reference sequence in the HOMD database were discarded. The chimeras and features with a frequency ≤10 or detected in a single sample were also removed. The resulting tables and sequences from all studies were merged by QIIME2’s merge and merge-seqs commands. Samples with <2000 reads were also removed. Taxonomic annotations were assigned to the representative sequences of each OTU using the HOMD database. For the factor groups containing samples with unknown metadata, the unknown sample was removed from the group before the downstream analyses.

### Defining the core microbiome

The core microbiome was determined based on the abundance and occurrence frequency of the OTUs. Two methods adapted from Shade and Stopnisek^30^ and Wu et al.^9^ were used. Considering the sequences involved in this integrated analysis were collected from studies targeted at different hypervariable regions, close-referenced clustering at 97% identity were used to cluster sequences into OTUs. In addition, taxa defined at 100% sequence identity may increase the redundancy in the dataset^30^. Therefore, the core salivary microbiome was defined using the clustered OTUs at 97% identity. For the methods adapted from the study of Shade and Stopnisek^30^, samples were rarefied to 5000 reads. Both Bray–Curtis similarity and weighted UniFrac distance were used to determine the contribution in the percentage of the prospective core set to the overall beta diversity. For the method adapted from Wu et al.^9^, OTUs were filtered out with the mean relative abundance (MRA) greater than 0.1% and the presence in more than 75% of samples or 100% occupancy in more than ten studies. To investigate the bacteria–bacteria interactions in salivary microbial communities, the co-occurrence network was constructed using pairwise Spearman’s correlation based on relative abundance. The Spearman’s correlation was calculated using the *rcorr* function in the *Hmisc* R package^67^ and visualised by Cytoscape v3.8.2^68^. A correlation with Spearman’s correlation coefficient >0.5 or <−0.5 and *p* value <0.01 is considered statistically robust and shown in the network.

### Diversity measures in R

Samples with >2000 reads were retained and processed with four normalisations: (1) Rarefying samples to 5000 (Rarefaction, RAR); (2) Samples were rarefied to 5000 and converted to relative abundance (Rarefied Total-sum Scaling, RRA); (3) Samples were converted to relative abundance directly (Total-sum scaling, TSS); (4) As described by Romano et al.^69^, zeros were added to data through the count zero multiplicative approach using the *cmultRepl* function of the *zCompostions* package^70^ in R (Centred Log-ratio, CLR).

The alpha diversity of all samples grouped by studies were calculated in the form of Chao1, Shannon and Simpson’s diversity indices. The beta diversity was assessed at different taxonomic levels, including OTU, species, genus, family, order, class and phylum level. For the OTU level, the beta diversity of data processed with the first three normalisations were determined using the weighted UniFrac distances and Bray–Curtis dissimilarities. Euclidean distances were calculated for all normalisations. At the other levels, Bray–Curtis dissimilarity was used for the first three normalisations, while Euclidean distances were combined with all the normalisations. Permutational multivariate analysis of variance (PERMANOVA) using the *adonis2* function of *vegan* package^71^ with 999 permutations was conducted to investigate the statistical differences caused by different factors, adjusting for the study.

### Differential abundance analyses

#### Random Forest

The normalised abundance of taxa in the phylum, class, order, family, genus, species and OTU level were classified against each provided metadata categories to determine which factor has the largest effect on the salivary microbiota. A random forest classifier was created in R using the *randomForest* package^72^ with default parameters. We used the *randomForest* (importance = TRUE, proximity = TRUE) function to generate the classification model for seven categories. For four categories that have the random forest classifier with an average error rate lower than 20%, including the hypervariable region (5.0%), geographic location (10.4%), tobacco usage (12.7%), sample type (13.8%), differential taxa were defined using cross-validation. Cross-validation was performed by the *rfcv()* function for selecting appropriate features. The *varImpPlot* function was used to show the importance of features in the classification. The importance of features and the cross-validation curve were visualised by using the *ggplot2* package^73^ in R.

#### Analysis of compositions of microbiomes with bias correction (ANCOM-BC)

ANCOM-BC^74^ were performed to identify the taxa with different relative abundance between Chinese and Western samples. Function ANCOM-BC were used with Holm–Bonferroni false discovery rate correction and other default parameters. The hypervariable regions used by different studies were used as the covariate.

### Functional prediction

Microbial metagenomes were inferred from 16S rRNA gene-based bacterial profiles, and the functional prediction were conducted based on the Kyoto Encyclopaedia of Gene and Genomes (KEGG) database^75^ using the default pipeline in Phylogenetic Investigation of Communities by Reconstruction of Unobserved States 2 (PICRUSt2)^56^. The ANCOM-BC analysis was used to identify the differential abundant KEGG pathways by geographic location, adjusting for hypervariable regions. At the same time, a random forest model was established for distinguishing Chinese and Western samples, and the importance of pathways was measured using mean decreased accuracy. Spearman’s correlation was performed to assess the relationship between the relative abundance of differential pathways and genera. The significant correlations were visualised using the *corrplot* package^76^ in R.

### Comparison between Chinese and Western people in an independent cohort

Saliva samples were collected from 26 participants (aged 20–60 years) recruited for a wine assessment experiment and consisted of 13 Chinese and 13 Western wine experts. The study was approved by the Office for Research Ethics and Integrity of the University of Melbourne (Ethics ID: 1852616). Each group had six female panelists and seven male panelists. The Western panelists were defined as people who have lived in Australia for more than ten years. Chinese panelists were defined as people who were born in China and had lived in Australia for no more than 18 months. Bacteria genomic DNA was extracted from human saliva using QIAGEN® MagAttract® PowerSoil® DNA KF Kit^77^ and subjected to 16S rRNA amplicon sequencing on the Illumina platform following the Earth Microbiome Project protocols (https://earthmicrobiome.org/protocols-and-standards/16s/). The raw data were available in NCBI Sequence Read Archive, with accession number PRJNA786805.

The raw sequences were firstly processed using the same pipeline in the integrated analysis as described in section 2. Due to benefits of finer resolution, defining taxa at 100% identity of sequencing read rather than 97% identity is increasing promoted^78,79^. To investigate the potential differences between Chinese and Western participants in the cohort with better sensitivity and specificity^78^, we further denoised the raw sequencing reads zero-radios OTUs (ZOTUs) by UNOISE3 pipeline^80^, and taxonomically classified the ZOTUs by classifiers trained on the full-length 16S rRNA gene SILVA v138^81^ database and eHOMD v15.22^66^, respectively. For validating the prevalence of the core we defined from the integrated analysis in this independent cohort, the affiliation between each ZOTUs and the originating OTU was determined using a customised code adapted from Stopnisek and Shade^82^ and available at https://github.com/XINWEIR/SalivaryMicrobiome_MetaAnalysis. The relative abundance of taxa at the genus level in this cohort was used as the test set for the random forest model trained using the genus-level OTU assignment information in the integrated analysis.

## Supplementary information

Supplementary Information

Supplementary Table 3

Supplementary Table 4

Supplementary Table 5

Supplementary Table 6

Supplementary Table 7

Supplementary Table 8

Supplementary Table 9

## Data Availability

The sequencing data supporting the conclusion of the integrated analysis in this article are available in publicly accessible databases (full details can be found in Supplementary Table 2). The sequencing data generated and/or analysed during the current study are available in the NCBI Bioproject repository, PRJNA786805 (https://www.ncbi.nlm.nih.gov/bioproject/786805).

## Code availability

Original scripts generated during the current study are available in Github (https://github.com/XINWEIR/SalivaryMicrobiome_MetaAnalysis).
