# Supplementary material for: The salivary microbiome shows a high prevalence of core bacterial members yet variability across human populations
Source: NPJ Biofilms Microbiomes. 2022 Oct 20;8:85. doi: 10.1038/s41522-022-00343-7 (PMC9584946; doi:10.1038/s41522-022-00343-7)
Supplement: Supplementary file 1 — Supplementary Information [file 41522_2022_343_MOESM1_ESM.pdf]

Supplementary information:

The salivary microbiome shows a high prevalence of core bacterial members yet variability across human populations

Xinwei Ruan[1], Jiaqiang Luo[1], Pangzhen Zhang[1] and Kate Howell[1, *]

[1]School of Agriculture and Food, Faculty of Veterinary and Agricultural Sciences, University of Melbourne, Parkville 3010, Australia

Xinwei Ruan: ORCID: 0000-0002-1963-3807

Jiaqiang Luo: ORCID: 0000-0001-6459-3309

Pangzhen Zhang: ORCID: 0000-0002-9794-2269

* Corresponding author. khowell@unimelb.edu.au; ORCID: 0000-0001-6498-0472

The supplementary information contains two parts:

1. **Supplementary Figures**
2. **Supplementary Tables**

Supplementary Figures

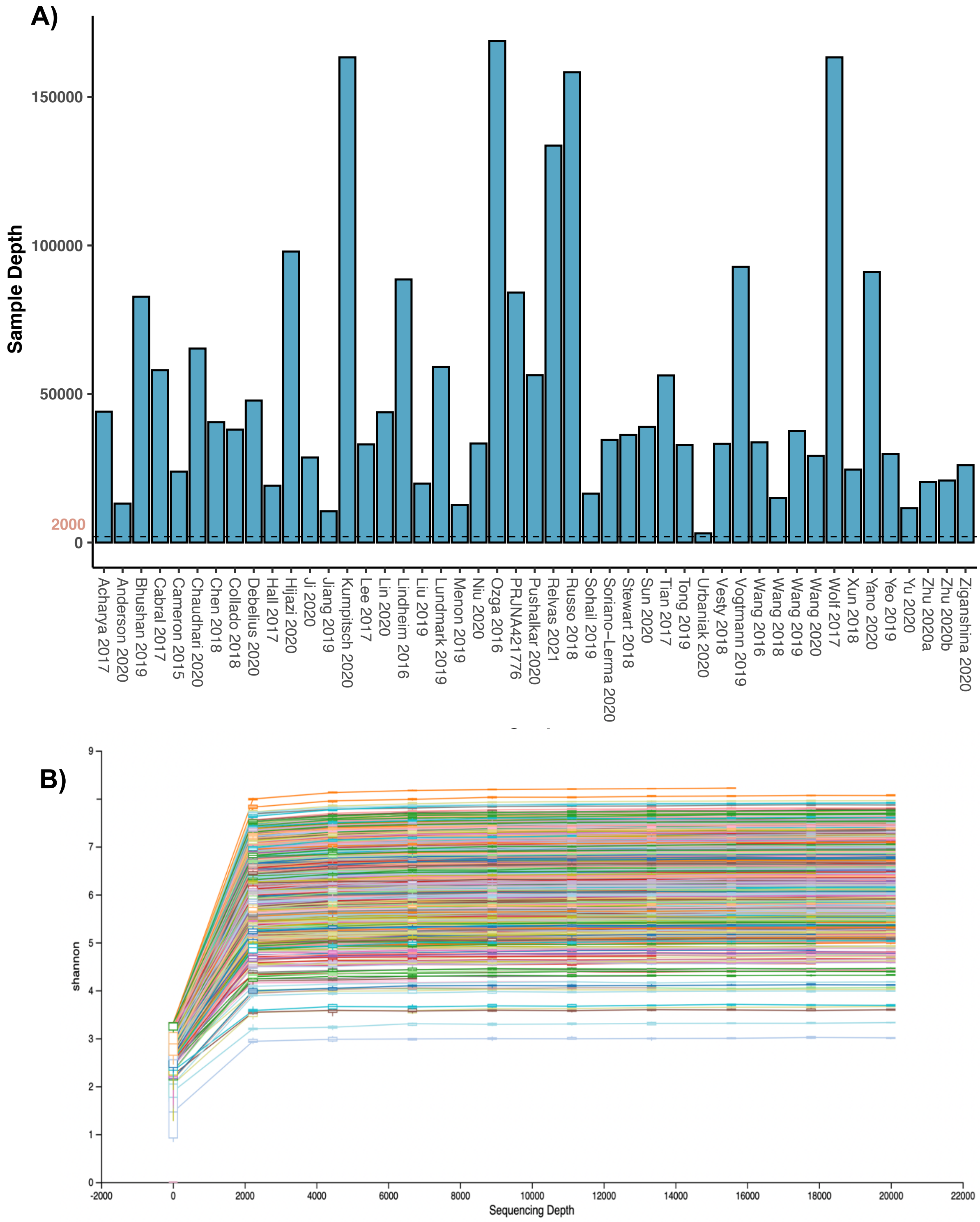

Supplementary Figure 1 Average sequencing Depth and rarefaction curve for the whole 16S rRNA dataset. A) Mean read number of samples from each study. The dash line indicates that all samples below this depth (depth = 2,000) have been removed. **B)** The rarefaction curve reflects the increase of sample's Shannon index with sequencing depth. The curve was basically stabilised at sequencing depth = 2000.

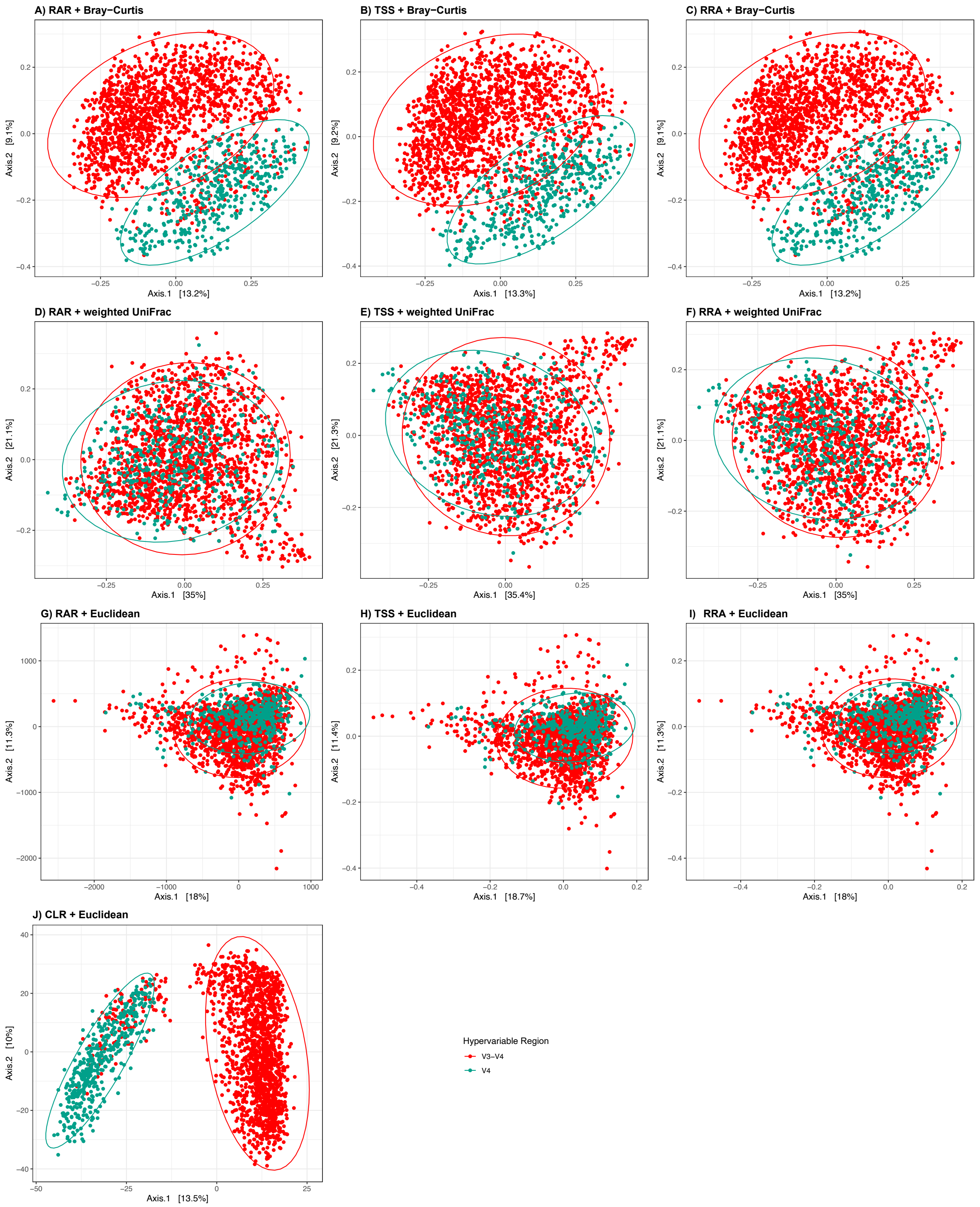

Supplementary Figure 2 The effect of different combinations between normalisations and distance matrices on reducing the impact of hypervariable regions in the PCoA plot. PCoA plots showing Bray-Curtis dissimilarity **(A-C)**, weighted uniFrac distance **(D-F)**, Euclidean distance **(G-J)** under rarefaction (RAR) **(A, D, G)**, total-sum scaling (TSS) **(B, E, H)**, rarefied relative abundance transformation (RRA) **(C, F, I)** and centred log-ratio transformation **(J)**. Percentage of variances explained by the first two principal coordinates are shown on the axes.

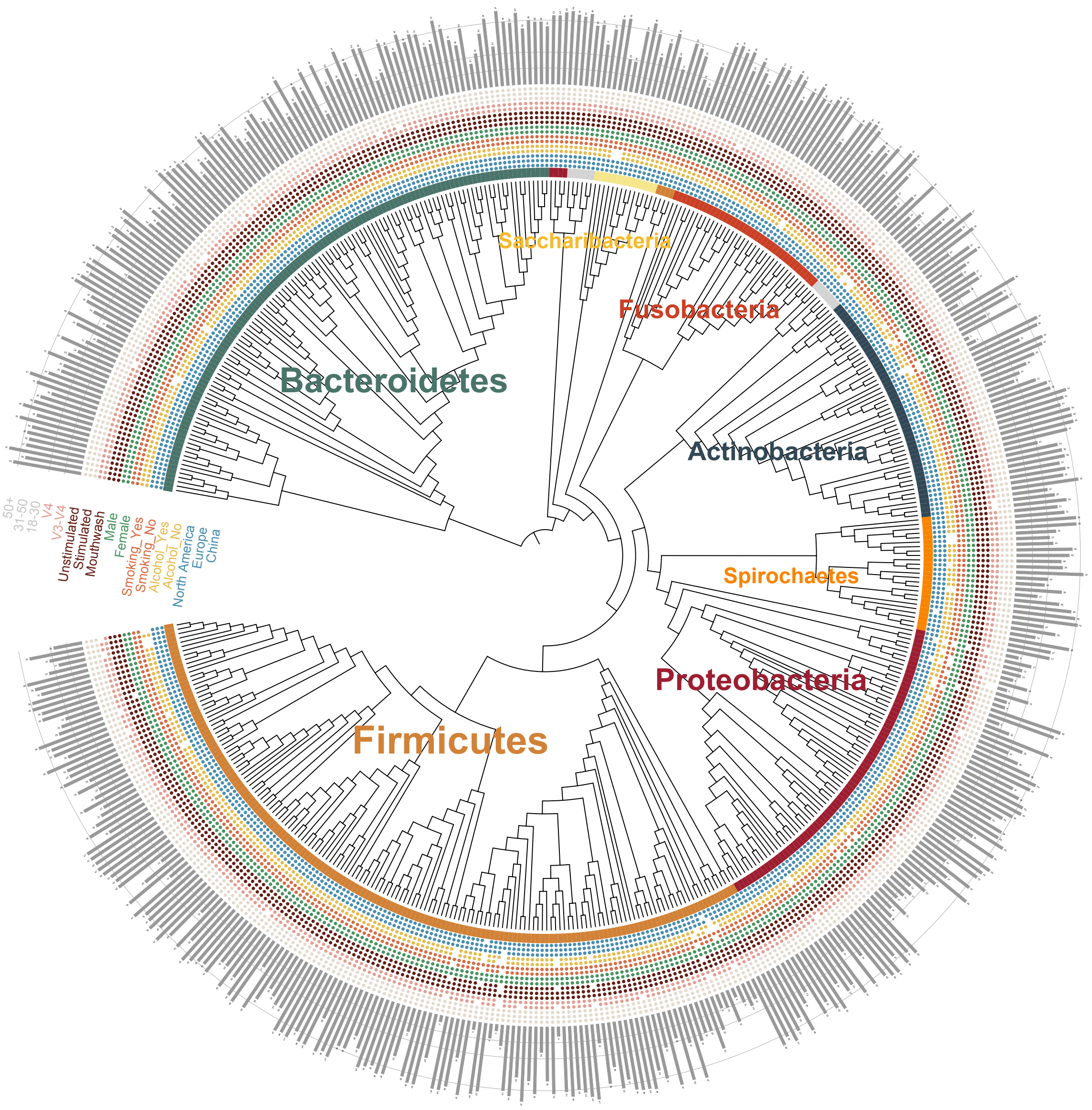

Supplementary Figure 3 Phylogenetic tree showing the presence and absence of top 500 OTUs with highest mean relative abundance. The colour strips on the innermost ring indicates which phylum the OTUs belong to. The presence of coloured circles on the 17 rings in the middle indicate that an OTU was found in a specific level of a sub-group. The grey bars on the outermost layer represent how many studies that an OTU presented. The scale lines are used to highlight the number of 10, 20 and 40.

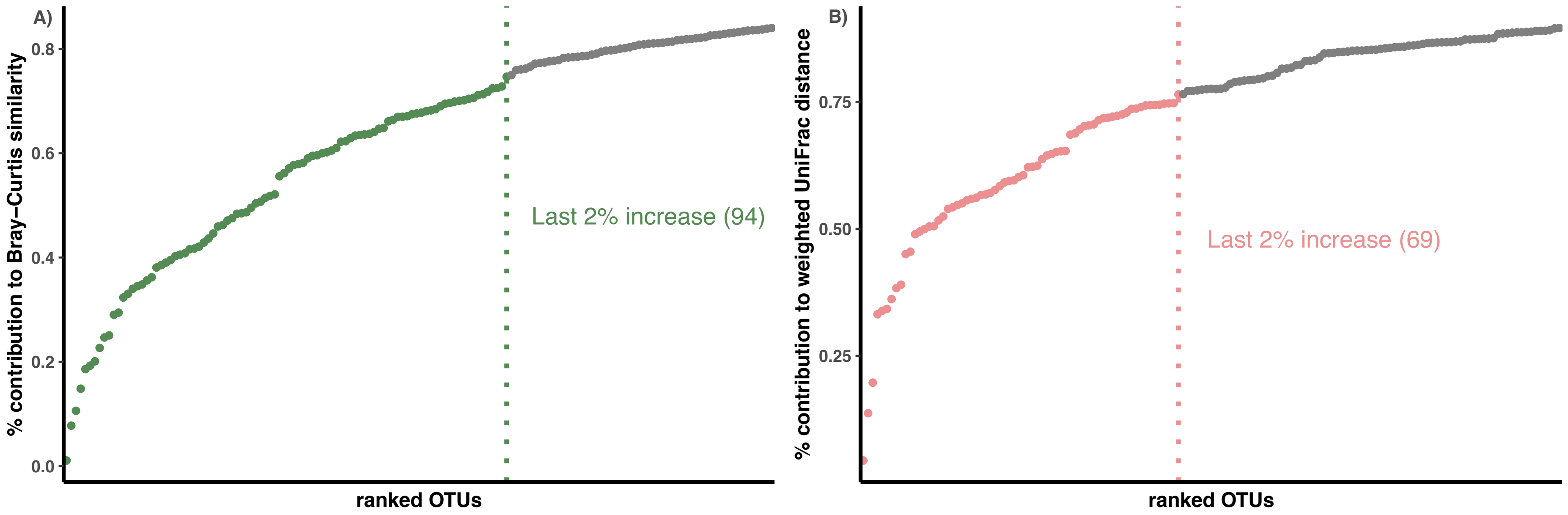

Supplementary Figure 4 The percentage contribution of the top-ranked OTUs to the beta-diversity of the dataset. The beta-diversity is calculated for the whole dataset and for only the top-ranked OTUs using both Bray-Curtis similarity (A) and weighted uniFrac distance (B). The percentage contribution of top-ranked OTUs is calculated by dividing the beta-diversity among top-ranked OTUs using the beta-diversity of the whole dataset. The dash lines indicate the last points at which the increase on the contribution is 2%. All OTUs before this point (the point itself was also included) were defined as “core”.

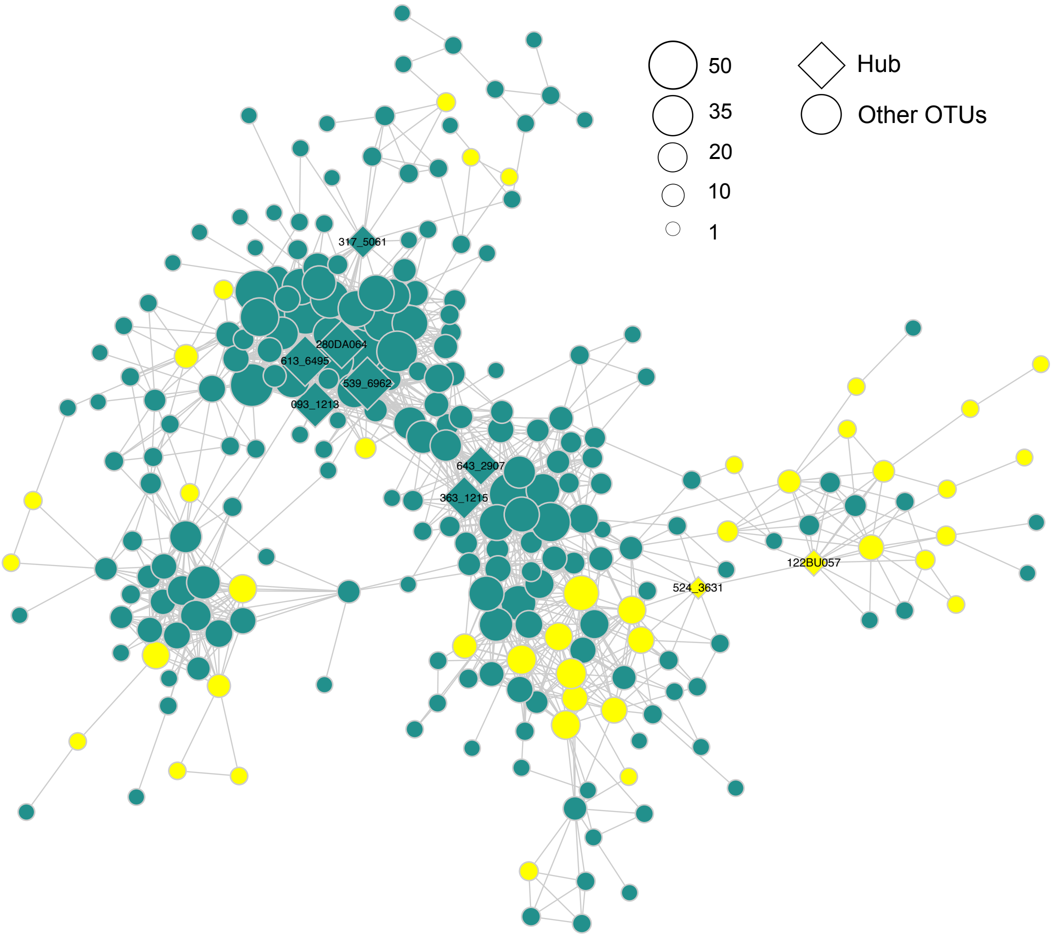

Supplementary Figure 5 Bacterial co-occurrence network verifies the role of identified core salivary microbiome members. Small modules with less than seven nodes were not displayed. The size of nodes is proportioned to the connectivity of nodes (node degree). Core OTUs from Figure 4 are indicated as yellow, while rare OTUs are in green. The edges between nodes represent the strength of the correlation (Spearman's correlation coefficient, $\rho \geq 0.5$, FDR adjusted $p < 0.01$). The shape of "hub" OTUs are indicated as squares with the OTU name displayed.

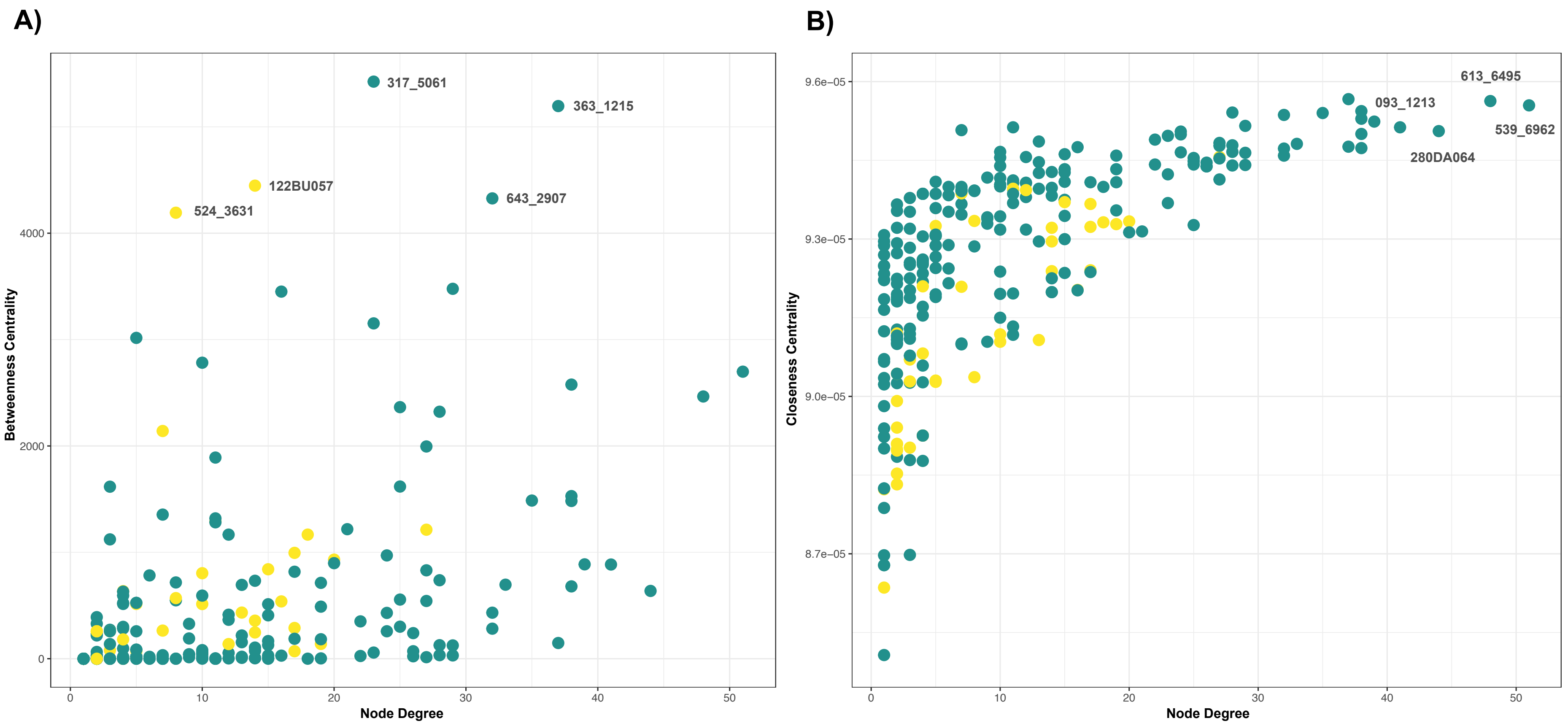

Supplementary Figure 6 The Betweenness and Closeness centrality of OTUs involved in the network analysis. The "hub" OTUs were identified as OTUs with either high connectivity (node degree) or centrality (betweenness **(A)** and closeness **(B)** centrality). The accession number of "hub" OTUs are indicated. The core OTUs are marked in yellow, while the rare OTUs were coloured in green.

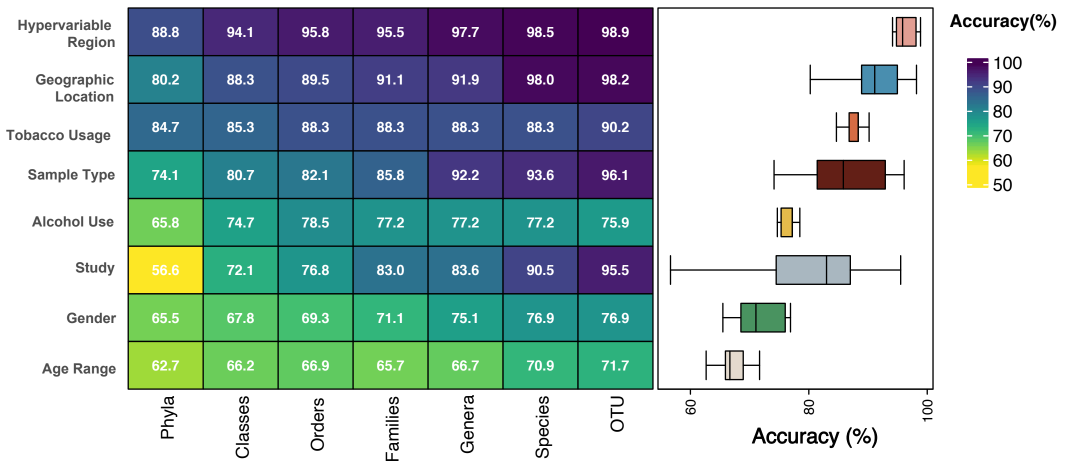

Supplementary Figure 7 Classification accuracy (%) for the random forest models conducted with samples grouped by eight different categories. The random forest models were built using the functions of the Caret R package. Five-fold cross-validation was performed; 75% and 25% of samples were used for training and testing.

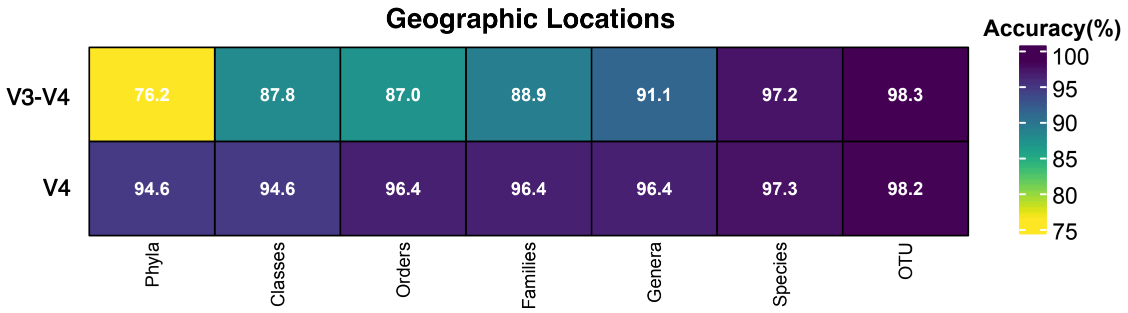

Supplementary Figure 8 Classification accuracy (%) for the random forest models constructed for samples within each hypervariable region grouped by geographic locations. The random forest models were built using the functions of the Caret R package. Five-fold cross-validation was performed; 75% and 25% of samples were used for training and testing.

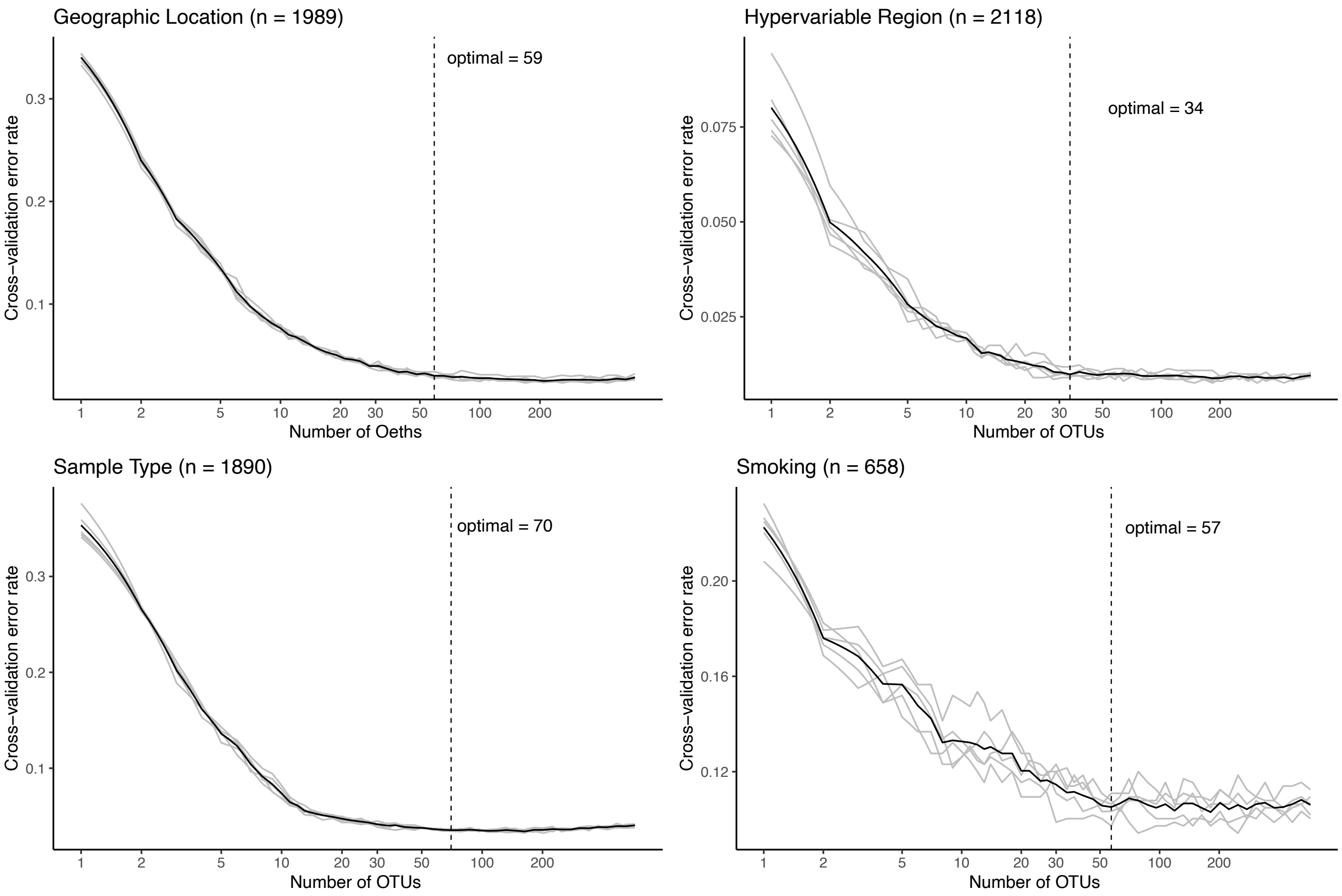

Supplementary Figure 9 The optimal number for defining the biomarker OTUs of four categories. The contribution of the OTUs used to differentiate the levels in each category on ten-fold cross-validation. The OTUs were ranked in the order of importance. The dash lines represent the point at which the curve starts to become overall stable.

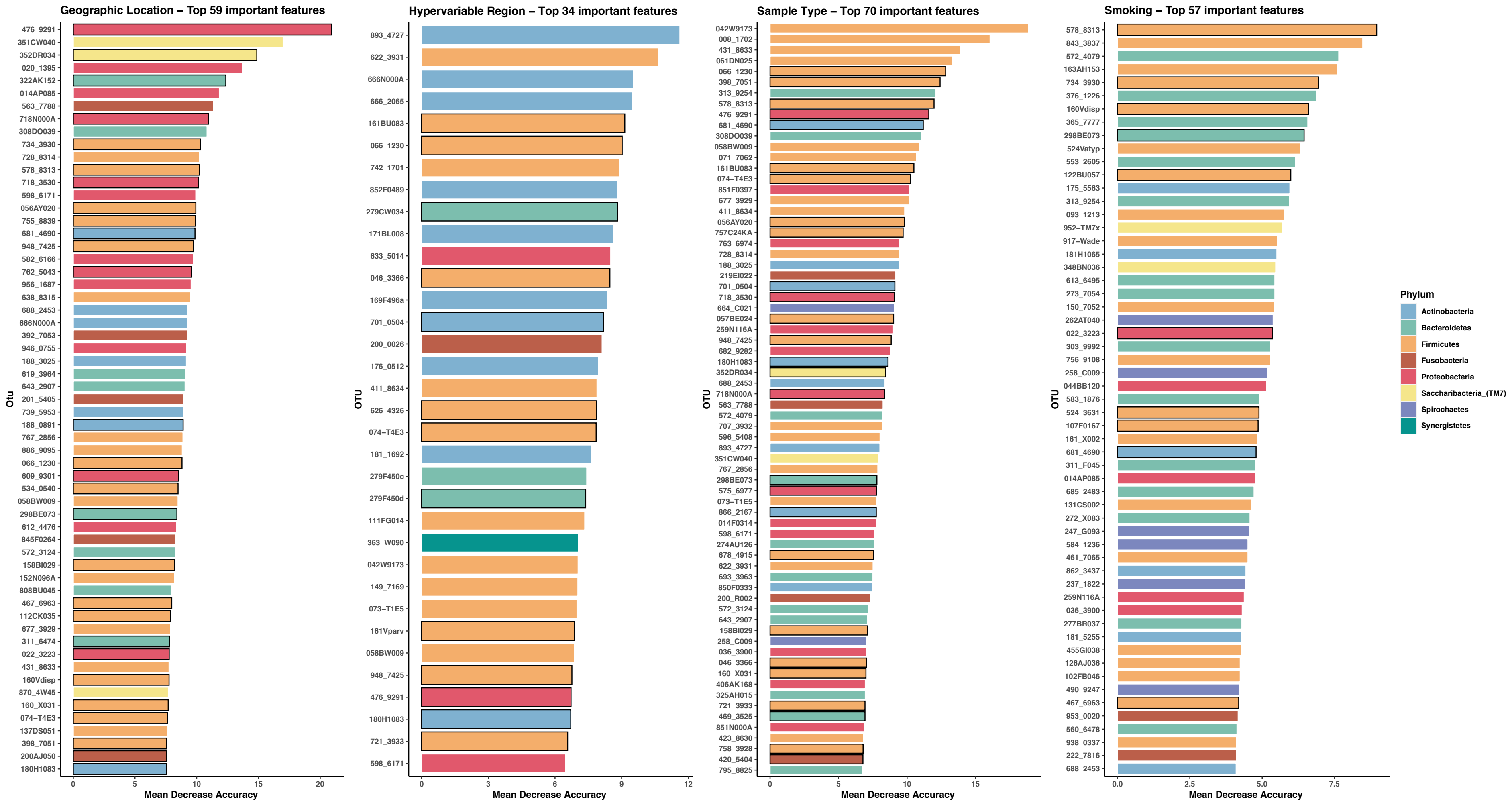

Supplementary Figure 10 The top important OTUs identified by Random Forest classification model established by the relative abundance of human salivary microbiota. The differential OTUs defined were ranked in descending order of their importance (Mean Decrease Accuracy). The colour of bars reflects the phylum level information of OTUs. The Mean Decrease Accuracy bar of core OTUs were marked with bolded black borders

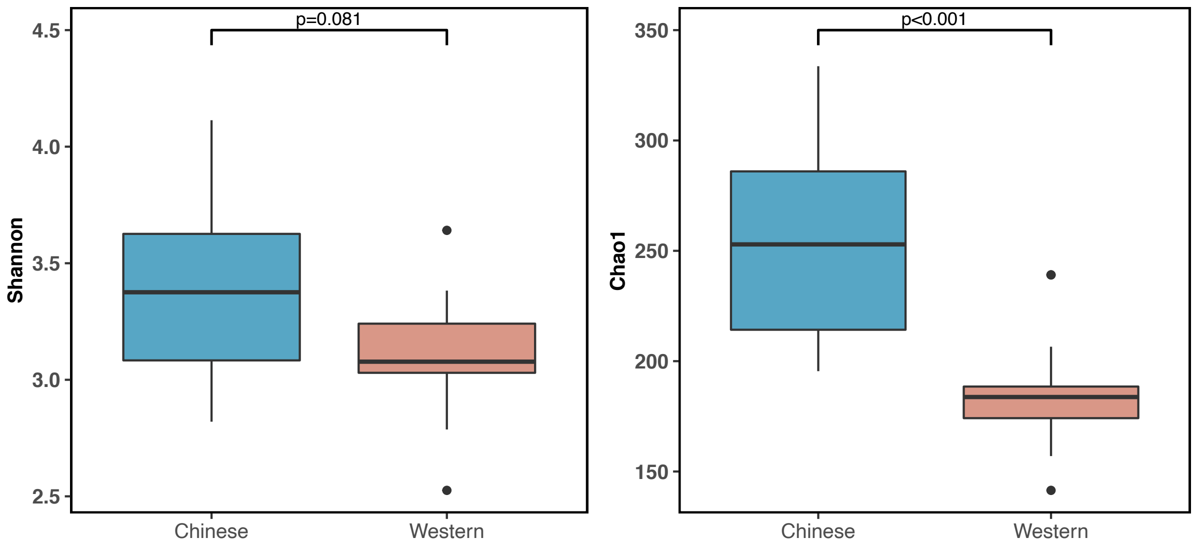

Supplementary Figure 11 Comparison of salivary microbial alpha diversity between the Chinese and Western samples. The alpha-diversity is calculated by Shannon (p = 0.081, Wilcoxon rank-sum test) and Chao1 index (p< 0.001, Wilcoxon rank-sum test), using the OTUs generated from OTU picking.

Supplemental Tables

Supplementary Table 1 Search Terms used on Medline, EMBASE, and Web of Science.

TERMS	Medline and EMBASE		Web of Science
**Saliva or Oral**	**Map Term to Subject Heading**	Saliva/	Saliva* or oral near/5 (microbiome OR microbiota OR bacteria*)
	OR		
	Keywords	Saliva* or oral adj5((microbe* or microbiome* or microbiota* or bacteria* or microbial) or Microbiota/ or exp Bacteria/)	
AND			
**Microbiome**	**Map Term to Subject Heading**	Microbiota/ or exp Bacteria/	Microbe* OR microbiome* OR microbiota* OR bacteria* OR microbial
	OR		
	Keywords	microbe* or microbiome* or microbiota* or bacteria* or microbial	
AND			
**16S rRNA amplicon sequencing**	**Map Term to Subject Heading**	RNA, Ribosomal, 16S/	"RNA, Ribosomal, 16S" OR "RNA 16S" OR 16S
	OR		
	Keywords	16S	

Supplementary Table 2 Studies included after large-scale literature searches that met all the inclusion criteria.

Study	Reference	Database	Accession Number	Location	Hypervariable Region	Number of Samples	Sample Type
Acharya 2017	1	SRA	PRJNA323410	India	V3-V4	49	Unstimulated
Anderson 2020	2	SRA	PRJNA577839	Europe	V3-V4	36	Stimulated
Bhushan 2019	3	SRA	SRP125370	India	V3-V4	6	Other
Cabral 2017	4	SRA	PRJNA380250	United States	V1-V2/V4-V5	8	Unstimulated
Cameron 2015	5	ENA	PRJEB9010	Europe	V3-V4	61	Stimulated
Chaudhari 2020	6	SRA	PRJNA438728	India	V3-V4	22	Mouthwash
Chen 2018	7	SRA	PRJNA321534	United States	V4	18	Unstimulated
Collado 2018	8	SRA	PRJNA361501	Europe	V3-V4	98	Other
Debelius 2020	9	ENA	PRJEB37445	China	V3-V4	436	Stimulated
Hall 2017	10	ENA	PRJEB11529	Canada	V3-V4	95	Stimulated
Hijazi 2020	11	SRA	PRJNA609244	Europe	V1-V2	13	Unstimulated
Ji 2020	12	SRA	PRJNA503603	China	V3-V4	24	Unstimulated
Jiang 2019	13	SRA	PRJNA495719	China	V3-V4	21	Unstimulated
Kumpitsch 2020	14	ENA	PRJEB37299	Europe	V4	11	Stimulated
Lee 2017	15	SRA	PRJNA386665	China	V4	127	Unstimulated

Lin 2020	[16]	SRA	PRJNA578492	China	V3-V4	14	Unstimulated
Lindheim 2016	[17]	SRA	PRJNA326866	Europe	V1-V2	20	Unstimulated
Liu 2019	[18]	SRA	PRJNA484857	China	V3-V4	15	Unstimulated
Lundmark 2019	[19]	ENA	PRJEB21767	Europe	V3-V4	47	Stimulated
Menon 2019	[20]	SRA	PRJNA586897	China	V3-V4	64	Unstimulated
Niu 2020	[21]	SRA	SRP113577	China	V4-V5	36	Unstimulated
Ozga 2016	[22]	SRA	PRJNA292800	United States	V4	18	Unstimulated
-	[23]	SRA	PRJNA421776	United States	V3-V4	85	Unknown
Pushalkar 2020	[24]	SRA	PRJNA602902	United States	V3-V4	119	Stimulated
Relvas 2021	[25]	SRA	PRJNA623352	Europe	V3-V4	17	Unstimulated
Russo 2018	[26]	SRA	PRJNA356414	Europe	V3-V4	10	Unstimulated
Sohail 2019	[27]	SRA	PRJNA587625	Qatar	V3-V4	73	Unstimulated
Soriano-Lerma 2020	[28]	SRA	PRJNA612815	Europe	V1-V3/V3-V4/V4-V5/V6-V8	22	Other
Stewart 2018	[29]	SRA	PRJNA413706	United States	V4	30	Stimulated
Sun 2020	[30]	SRA	PRJNA601054	China	V3-V4	27	Unstimulated
Tian 2017	[31]	SRA	PRJNA321349	United States	V3-V4	20	Stimulated

Tong 2019	[32]	SRA	PRJNA578951	China	V3-V4	23	Unstimulated
Urbaniak 2020	[33]	SRA	PRJNA539937	United States	V3-V4	13	Other
Vesty 2018	[34]	SRA	PRJNA421234	New Zealand	V3-V4	6	Unstimulated
Vogtmann 2019	[35]	Qiita	10823	United States	V4	138	Mouthwash
Wang 2016	[36]	SRA	PRJNA306560	China	V4	18	Stimulated
Wang 2018	[37]	SRA	PRJNA414355	China	V3-V4	36	Unstimulated
Wang 2019	[38]	SRA	PRJNA587078/	China	V3-V4	21	Unstimulated
Wang 2020	[39]	SRA	PRJNA556311	China	V3-V4	20	Unstimulated
Wolf 2017	[40]	ENA	PRJEB18476	Europe	V4	11	Stimulated
Xun 2018	[41]	SRA	PRJNA414682	China	V3-V4	20	Unstimulated
Yano 2020	[42]	SRA	PRJNA634162	United States	V4	75	Other
Yeo 2019	[43]	SRA	PRJNA515166	Malaysia	V3-V4	66	Unstimulated
Yu 2020	[44]	SRA	PRJNA542018	China	V4	10	Unstimulated
Zhu 2020a	[45]	SRA	PRJNA586723	China	V3-V4	60	Stimulated and unstimualted
Zhu 2020b	[46]	SRA	PRJNA534340	China	V3-V4	40	Stimulated and unstimualted
Ziganshina 2020	[47]	SRA	PRJNA598080	Europe	V3-V4	12	Unstimulated

Supplementary Table 3 The full metadata used in this study, including 2206 samples with unique accession numbers. **(Supplementary Table 3.xls)**

Supplementary Table 4 The influence of seven factors at seven taxonomic levels on human salivary microbial communities, measured by PERMANOVA with adonis2 function (permutation = 999). PERMANOVA models were adjusted for study. **(Supplementary Table 4.xls)**

Supplementary Table 5 Core OTUs of adults' saliva microbiome defined by **A)** method adapted from Wu et al. (2019) ("1"= yes, "0"=no), **B)** method adapted from Shade and Stopnisek (2019). **(Supplementary Table 5.xls)**

Supplementary Table 6 Genus with differential abundance between samples from Western and Chinese people identified by both ANCOM-BC and Random Forest model, adjusted for hypervariable regions sequenced. **(Supplementary Table 6.xls)**

Supplementary Table 7 Species with differential abundance between samples from Western and Chinese people identified by both ANCOM-BC and Random Forest model, adjusted for hypervariable regions sequenced. **(Supplementary Table 7.xls)**

Supplementary Table 8 KEGG pathways with differential abundance between samples from Western and Chinese people identified by both ANCOM-BC and Random Forest model, adjusted for hypervariable regions sequenced. **(Supplementary Table 8.xls)**

Supplementary Table 9 The differences between Chinese and Western participants in the independent cohort, measured by PERMANOVA with adonis2 function (permutation = 999). The PERMANOVA model was adjusted for the gender and age range of participants. **(Supplementary Table 9.xls)**

References

1 Acharya, A. *et al.* Salivary microbiome of an urban Indian cohort and patterns linked to subclinical inflammation. *Oral Dis.* **23**, 926-940, (2017).

2 Anderson, A. *et al.* Influence of the long-term use of oral hygiene products containing stannous ions on the salivary microbiome–a randomized controlled trial. *Sci. Rep.* **10**, 1-8, (2020).

3 Bhushan, B., Yadav, A., Singh, S. & Ganju, L. Diversity and functional analysis of salivary microflora of Indian Antarctic expeditionaries. *J. Oral Microbiol.* **11**, 1581513, (2019).

4 Cabral, D. J. *et al.* The salivary microbiome is consistent between subjects and resistant to impacts of short-term hospitalization. *Sci. Rep.* **7**, 11040, (2017).

5 Cameron, S. J. S., Huws, S. A., Hegarty, M. J., Smith, D. P. M. & Mur, L. A. J. The human salivary microbiome exhibits temporal stability in bacterial diversity. *FEMS Microbiol. Ecol.* **91**, (2015).

6 Chaudhari, D. S. *et al.* Gut, oral and skin microbiome of Indian patrilineal families reveal perceptible association with age. *Sci. Rep.* **10**, 5685, (2020).

7 Chen, C. *et al.* Oral microbiota of periodontal health and disease and their changes after nonsurgical periodontal therapy. *ISME J.* **12**, 1210-1224, (2018).

8 Collado, M. C. *et al.* Timing of food intake impacts daily rhythms of human salivary microbiota: a randomized, crossover study. *FASEB J.* **32**, 2060-2072, (2018).

9 Debelius, J. W. *et al.* Subspecies niche specialization in the oral microbiome is associated with nasopharyngeal carcinoma risk. *Msystems* **5**, e00065-00020, (2020).

10 Hall, M. W. *et al.* Inter-personal diversity and temporal dynamics of dental, tongue, and salivary microbiota in the healthy oral cavity. *NPJ Biofilms Microbiomes* **3**, 2, (2017).

11 Hijazi, K. *et al.* Oral bacterial diversity is inversely correlated with mucosal inflammation. *Oral Dis.* **26**, 1566-1575, (2020).

12 Ji, Y., Liang, X. & Lu, H. Analysis of by high-throughput sequencing: Helicobacter pylori infection and salivary microbiome. *BMC Oral Health* **20**, 84, (2020).

13 Jiang, Q., Liu, J., Chen, L., Gan, N. & Yang, D. The Oral Microbiome in the Elderly With Dental Caries and Health. *Front. Cell. Infect. Microbiol.* **8**, (2019).

14 Kumpitsch, C., Moissl-Eichinger, C., Pock, J., Thurnher, D. & Wolf, A. Preliminary insights into the impact of primary radiochemotherapy on the salivary microbiome in head and neck squamous cell carcinoma. *Sci. Rep.* **10**, 1-12, (2020).

15 Lee, W.-H. *et al.* Bacterial alterations in salivary microbiota and their association in oral cancer. *Sci. Rep.* **7**, 1-11, (2017).

16 Lin, M. *et al.* Saliva microbiome changes in patients with periodontitis with and without chronic obstructive pulmonary disease. *Front. Cell. Infect. Microbiol.* **10**, 124, (2020).

17 Lindheim, L. *et al.* The salivary microbiome in polycystic ovary syndrome (pcos) and its association with disease-related parameters: a pilot study. *Front. Microbiol.* **7**, 1270, (2016).

18 Liu, Y., Zhang, Q., Hu, X., Chen, F. & Hua, H. Characteristics of the salivary microbiota in cheilitis granulomatosa. *Med. Oral Patol. Oral Cir. Bucal.* **24**, e719-e725, (2019).

19 Lundmark, A. *et al.* Identification of salivary microbiota and its association with host inflammatory mediators in periodontitis. *Front. Cell. Infect. Microbiol.* **9**, 216, (2019).

20 Menon, R. *et al.* Long-term impact of oral surgery with or without amoxicillin on the oral microbiome-A prospective cohort study. *Sci. Rep.* **9**, 1-10, (2019).

21 Niu, C. *et al.* Pregnancy-Related Ecological Shifts of Salivary Microbiota and its Association with Salivary Sex Hormones. (2020).

22 Ozga, A. T. *et al.* Oral microbiome diversity among Cheyenne and Arapaho individuals from Oklahoma. *Am. J. Phys. Anthropol.* **161**, 321-327, (2016).

23 PRJNA421776. Exercise frequency is associated with the oral microbiota of student athletes and non-athletes, https://www.ncbi.nlm.nih.gov/bioproject/PRJNA421776 (2017).

24 Pushalkar, S. *et al.* Electronic Cigarette Aerosol Modulates the Oral Microbiome and Increases Risk of Infection. *iScience* **23**, 100884, (2020).

25 Relvas, M. *et al.* Relationship between dental and periodontal health status and the salivary microbiome: bacterial diversity, co-occurrence networks and predictive models. *Sci. Rep.* **11**, 929, (2021).

26 Russo, E. *et al.* Preliminary comparison of oral and intestinal human microbiota in patients with colorectal cancer: a pilot study. *Front. Microbiol.* **8**, 2699, (2018).

27 Sohail, M. U., Elrayess, M. A., Al Thani, A. A., Al-Asmakh, M. & Yassine, H. M. Profiling the oral microbiome and plasma biochemistry of obese hyperglycemic subjects in Qatar. *Microorganisms* **7**, 645, (2019).

28 Soriano-Lerma, A. *et al.* Influence of 16S rRNA target region on the outcome of microbiome studies in soil and saliva samples. *Sci. Rep.* **10**, 1-13, (2020).

29 Stewart, C. J. *et al.* Effects of tobacco smoke and electronic cigarette vapor exposure on the oral and gut microbiota in humans: a pilot study. *PeerJ* **6**, e4693, (2018).

30 Sun, X. *et al.* Alteration of salivary microbiome in periodontitis with or without type-2 diabetes mellitus and metformin treatment. *Sci. Rep.* **10**, 1-14, (2020).

31 Tian, N. *et al.* Salivary gluten degradation and oral microbial profiles in healthy individuals and celiac disease patients. *Appl Environ Microbiol* **83**, e03330-03316, (2017).

32 Tong, Y. *et al.* Oral microbiota perturbations are linked to high risk for rheumatoid arthritis. *Front. Cell. Infect. Microbiol.* **9**, 475, (2020).

33 Urbaniak, C. *et al.* The influence of spaceflight on the astronaut salivary microbiome and the search for a microbiome biomarker for viral reactivation. *Microbiome* **8**, 1-14, (2020).

34 Vesty, A. *et al.* Microbial and inflammatory-based salivary biomarkers of head and neck squamous cell carcinoma. *Clin. Exp. Dent. Res.* **4**, 255-262, (2018).

35 Vogtmann, E. *et al.* Comparison of oral collection methods for studies of microbiota. *Cancer Epidemiol. Biomark. Prev.* **28**, 137-143, (2019).

36 Wang, K. *et al.* Preliminary analysis of salivary microbiome and their potential roles in oral lichen planus. *Sci. Rep.* **6**, 22943, (2016).

37 Wang, T. *et al.* Chronic fatigue syndrome patients have alterations in their oral microbiome composition and function. *PLoS One* **13**, e0203503, (2018).

38 Wang, Q. *et al.* Oral microbiome in patients with oesophageal squamous cell carcinoma. *Sci. Rep.* **9**, 1-9, (2019).

39 Wang, X. *et al.* Microbial community analysis of saliva and biopsies in patients with oral lichen planus. *Front. Microbiol.* **11**, 629, (2020).

40 Wolf, A. *et al.* The salivary microbiome as an indicator of carcinogenesis in patients with oropharyngeal squamous cell carcinoma: A pilot study. *Sci. Rep.* **7**, 1-10, (2017).

41 Xun, Z., Zhang, Q., Xu, T., Chen, N. & Chen, F. Dysbiosis and Ecotypes of the Salivary Microbiome Associated With Inflammatory Bowel Diseases and the

Assistance in Diagnosis of Diseases Using Oral Bacterial Profiles. *Front. Microbiol.* **9**, (2018).

42 Yano, Y. *et al.* Comparison of Oral Microbiota Collected Using Multiple Methods and Recommendations for New Epidemiologic Studies. *Msystems* **5**, e00156-00120, (2020).

43 Yeo, L.-F., Aghakhanian, F. F., Tan, J. S., Gan, H. M. & Phipps, M. E. Health and saliva microbiomes of a semi-urbanized indigenous tribe in Peninsular Malaysia. *F1000Research* **8**, (2019).

44 Yu, F. Y. *et al.* Dysbiosis of saliva microbiome in patients with oral lichen planus. *BMC Microbiol.* **20**, 1-12, (2020).

45 Zhu, C., Yuan, C., Wei, F.-Q., Sun, X.-Y. & Zheng, S.-G. Comparative evaluation of peptidome and microbiota in different types of saliva samples. *Ann Transl Med* **8**, 686-686, (2020).

46 Zhu, C., Yuan, C., Wei, F. Q., Sun, X. Y. & Zheng, S. G. Intraindividual Variation and Personal Specificity of Salivary Microbiota. *J. Dent. Res.* **99**, 1062-1071, (2020).

47 Ziganshina, E. E. *et al.* Comparison of the Microbiota and Inorganic Anion Content in the Saliva of Patients with Gastroesophageal Reflux Disease and Gastroesophageal Reflux Disease-Free Individuals. *Biomed Res. Int.* **2020**, 2681791, (2020).
